# Human APOBEC3B promotes tumor development *in vivo* including signature mutations and metastases

**DOI:** 10.1016/j.xcrm.2023.101211

**Published:** 2023-10-04

**Authors:** Cameron Durfee, Nuri Alpay Temiz, Rena Levin-Klein, Prokopios P. Argyris, Lene Alsøe, Sergio Carracedo, Alicia Alonso de la Vega, Joshua Proehl, Anna M. Holzhauer, Zachary J. Seeman, Xingyu Liu, Yu-Hsiu T. Lin, Rachel I. Vogel, Rocio Sotillo, Hilde Nilsen, Reuben S. Harris

**Affiliations:** 1 Department of Biochemistry and Structural Biology, University of Texas Health San Antonio, San Antonio, TX 78229, USA; 2 Institute for Health Informatics, University of Minnesota, Minneapolis, MN 55455, USA; 3 Masonic Cancer Center, University of Minnesota, Minneapolis, MN 55455, USA; 4 Division of Oral and Maxillofacial Pathology, College of Dentistry, Ohio State University, Columbus, OH 43210, USA; 5 Department of Microbiology, Institute of Clinical Medicine, University of Oslo, 0318 Oslo, Norway; 6 Department of Microbiology, Oslo University Hospital, 0424 Oslo, Norway; 7 Division of Molecular Thoracic Oncology, German Cancer Research Center (DKFZ), 69120 Heidelberg, Germany; 8 Translational Lung Research Center Heidelberg (TRLC), German Center for Lung Research (DZL), 69120 Heidelberg, Germany; 9 Department of Obstetrics, Gynecology, and Women’s Health, University of Minnesota, Minneapolis, MN 55455, USA; 10 Howard Hughes Medical Institute, University of Texas Health San Antonio, San Antonio, TX 78229, USA

**Keywords:** APOBEC3B, cancer, DNA mutagenesis, lymphoma, murine tumor model, tumor heterogeneity

## Abstract

The antiviral DNA cytosine deaminase APOBEC3B has been implicated as a source of mutation in many cancers. However, despite years of work, a causal relationship has yet to be established *in vivo*. Here, we report a murine model that expresses tumor-like levels of human APOBEC3B. Animals expressing full-body APOBEC3B appear to develop normally. However, adult males manifest infertility, and older animals of both sexes show accelerated rates of carcinogenesis, visual and molecular tumor heterogeneity, and metastasis. Both primary and metastatic tumors exhibit increased frequencies of C-to-T mutations in TC dinucleotide motifs consistent with the established biochemical activity of APOBEC3B. Enrichment for APOBEC3B-attributable single base substitution mutations also associates with elevated levels of insertion-deletion mutations and structural variations. APOBEC3B catalytic activity is required for all of these phenotypes. Together, these studies provide a cause-and-effect demonstration that human APOBEC3B is capable of driving both tumor initiation and evolution *in vivo*.

## Highlights

• Expression of the human DNA deaminase APOBEC3B in mice causes male sterility
• Catalytically active APOBEC3B drives accelerated rates of tumor development
• APOBEC3B-expressing tumors exhibit signature C-to-T mutations in TCW motifs
• APOBEC3B signature enrichment associates positively with increased levels of indels

## Graphical abstract

**Figure:**
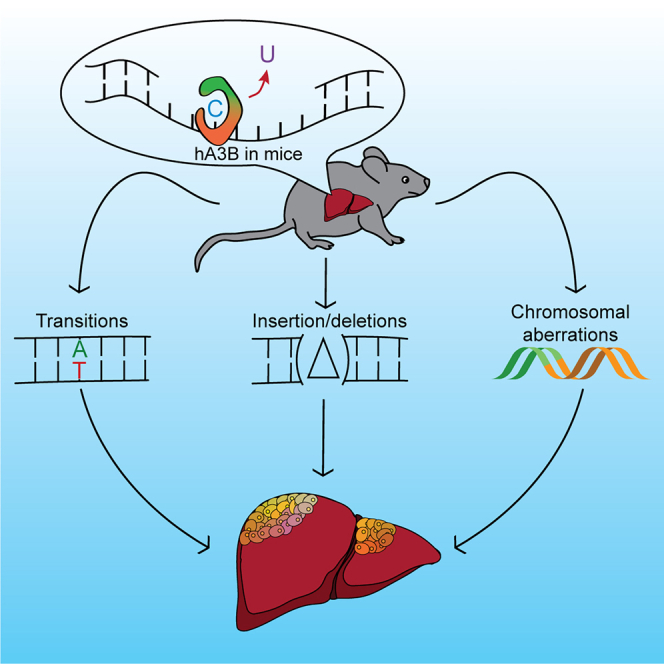

## Teaser

Expression of the human antiviral DNA cytosine deaminase APOBEC3B in mice accelerates tumor development and promotes tumor heterogeneity including overt phenotypic differences, thousands of signature single base substitution mutations (SBS2), and larger-scale chromosomal aberrations including insertion-deletion mutations and structural variations.

## Introduction

Cancer development and progression are evolutionary processes driven by mutations and further fueled by epigenetic alterations and environmental factors (reviewed by Hanahan,^1^ Persi et al.,^2^ and Reiter et al.^3^). Major advances over the past decade in genome sequencing and computational technologies have provided an unprecedented view of the entire landscape of genomic alterations that occur in cancer. These technologies have also yielded new information on the many oncoproteins and tumor suppressors that contribute to over 50 different human cancer types. Another profound advance enabled by these technologies is the capacity to extract distinct mutation signatures from otherwise complex montages of mutational events in single tumors (reviewed by Koh et al.,^4^ Alexandrov et al.,^5^ and Saini and Gordenin^6^). Upon extension to large numbers of tumors, the abundance of each distinct signature becomes starkly apparent and, taken together with chemical, biological, and genetic information, yields inferences to the most likely etiologic source (endogenous or exogenous) of the DNA damage that led to the observed signature. A few of many robust examples to date include spontaneous, water-mediated deamination of methyl-C to T in CG motifs (COSMIC single base substitution signature 1 [SBS1]), C-to-T mutations in di-pyrimidine motifs caused by A insertion opposite UV light-catalyzed pyrimidine dimers (SBS7), and APOBEC-catalyzed C-to-U deamination events in TC motifs leading to C-to-T and C-to-G mutations (SBS2 and SBS13, respectively).^5^

The human APOBEC family of polynucleotide C-to-U deaminase enzymes is comprised of apolipoprotein B mRNA editing catalytic subunit 1 (APOBEC1; the family namesake), activation-induced cytidine deaminase (AICDA; popularly called AID), and seven distinct APOBEC3 enzymes (A3A, B, C, D, F, G, and H; reviewed by Harris and Dudley,^7^ Pecori et al.,^8^ and Swanton et al.^9^). APOBEC1 functions in mRNA editing, AID in antibody gene diversification, and A3A–H in virus restriction. Although most of these enzymes preferentially deaminate TC motifs in single-stranded (ss)DNA, a number of studies have converged on A3A and A3B as the major sources of APOBEC signature mutations in cancer (see Carpenter et al.^10^ and Petljak et al.^11^ and references therein). Specifically, expression of A3A or A3B triggers an abundance of APOBEC signature mutations in human cells, and CRISPR-mediated gene knockouts lower the capacity of cancer cell lines to accumulate both SBS2 and SBS13 mutation signatures.^10^ Summaries of relevant literature including clinical correlations have been published (reviewed by Salas-Briceno et al.^12^ and Law et al.^13^).

A major obstacle in assessing the overall impact of A3A and A3B in cancer is a lack of appropriate murine models. Mice encode homologs of human APOBEC1 and AID but lack direct equivalents of human A3A and A3B (i.e., mice encode only a single Apobec3 protein with a domain organization not found in humans). Moreover, murine Apobec3 is cytoplasmic, and APOBEC signature mutations as defined above do not occur naturally in mice (reviewed by Salas-Briceno et al.^12^). However, recent studies have begun to overcome this obstacle by developing murine model systems to study mutagenesis by human A3A and A3B. First, a transgenic line that expresses low levels of human A3A has no cancer phenotypes alone but is capable of enhancing the penetrance of Apc^Min^-driven colorectal tumors and causing an accumulation of SBS2 (but not SBS13) signature mutations.^13^ Second, hydrodynamic delivery of human A3A into murine hepatocytes, coupled to liver regeneration by selecting for Fah function, results in hepatocellular carcinoma development within 6 months.^13^ Importantly, liver tumor formation in this model system requires A3A catalytic activity.^14^ However, expression of human A3A is rapidly selected against and lost early in hepatocellular carcinoma development, which limits the potential for longer-term studies on tumor evolution. Moreover, A3B expression is aphenotypic over the same duration in the Fah system.^13^ Last, low levels of human A3B expressed constitutively in mice from the endogenous *Rosa26* promotor cause no overt tumor phenotypes and no detectable APOBEC signature mutations^15^ (hereafter, this low A3B expression model is called *R26-A3B*).

**Figure 1:**
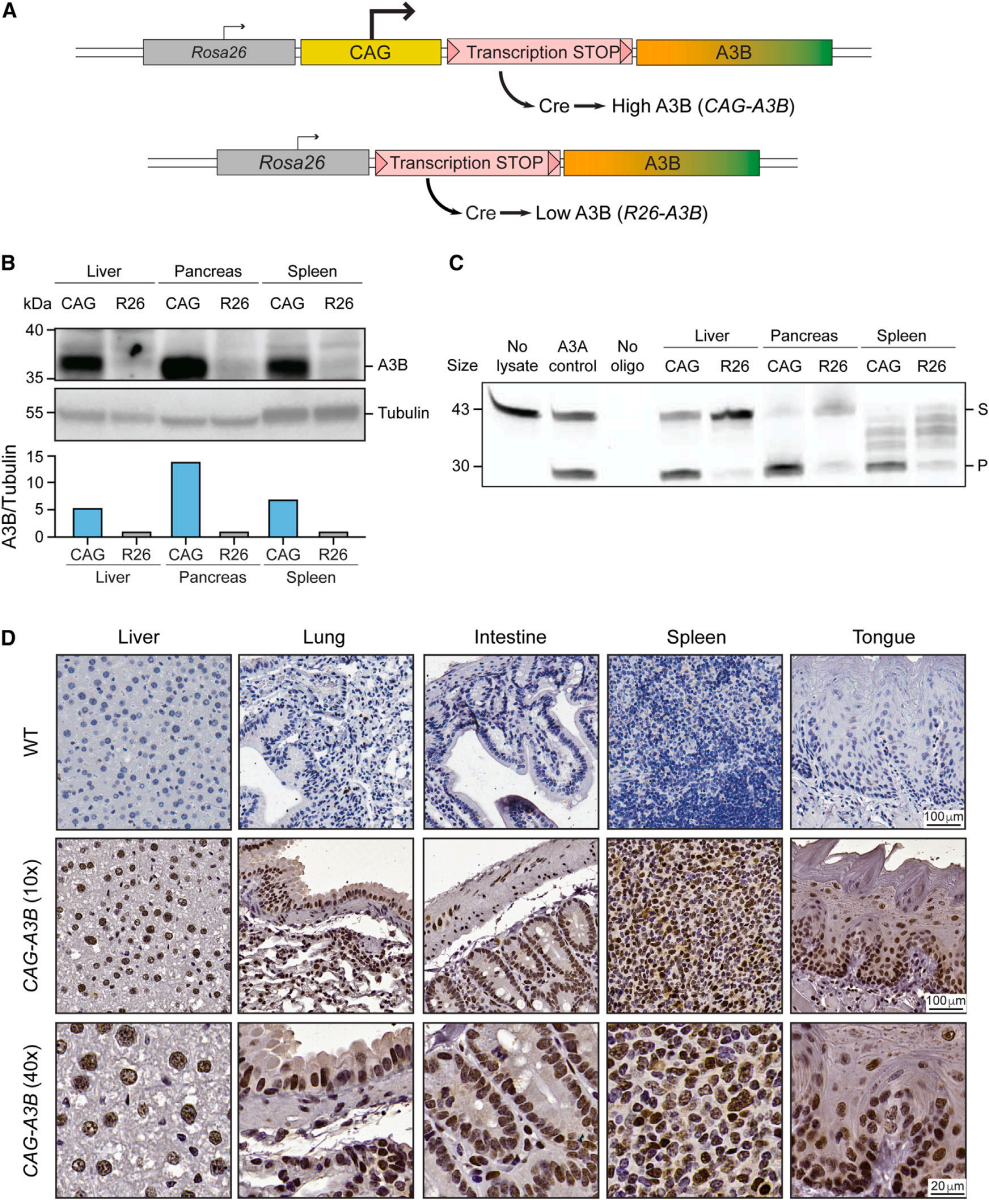
Murine models for inducible expression of human A3B (A) Schematics of our two different *Rosa26* knockin *A3B* minigene constructs. Human A3B expression at high (*CAG-A3B*) or low (*R26-A3B*) levels, respectively, occurs after Cre-mediated excision of the *loxP* (pink triangle)-flanked transcription stop cassette. (B and C) Immunoblot and ssDNA deaminase activity of human A3B protein expressed in the indicated tissues from *CAG-A3B* and *R26-A3B* animals. Tubulin provides a loading control, and recombinant A3A is a positive control for activity (S, substrate; P, product). Normalized A3B signal quantification relative to tubulin is shown below the immunoblot. (D) Anti-A3B IHC staining of representative tissues from WT and *CAG-A3B* mice (40× magnifications are enlargements of regions of the corresponding 10× images). See also Figure S1.

Thus, although A3B has been implicated in driving tumor progression and evolution in humans, published studies have yet to recapitulate these effects in mice. Establishing such a model is important for examining A3B’s role as a cancer driver and conducting additional studies on the underlying mechanisms and potential therapies. We have therefore created a murine model for inducible expression of human A3B. In these animals, a human *A3B* minigene is integrated into the *Rosa26* locus downstream of the *Rosa26* promoter, a stronger heterologous *CAG* promoter, and a strong transcription stop cassette flanked by *loxP* sites (*Rosa26::CAG-LSL-A3Bi*; schematic in Figure 1A). In this system, Cre-mediated removal of the transcription stop cassette results in strong A3B expression levels that recapitulate protein amounts reported in many human cancers (hereafter called *CAG-A3B*). Young animals show no overt phenotypes except that males are sterile. Older *CAG-A3B* mice of both sexes develop tumors, predominantly blood and liver cancers, an average of 5.2 months earlier than wild-type animals. A subset of *CAG-A3B* animals also show evidence for metastasis. Both primary and metastatic tumors manifest a pronounced APOBEC3 mutation signature (SBS2), which also associates with an elevated occurrence of structural variations, including small insertion and deletion (indel) mutations and larger-scale chromosomal aberrations. Moreover, A3B catalytic activity is required for these cancer phenotypes, as animals expressing an otherwise isogenic A3B-E255A protein exhibit near-normal lifespans and rates of tumor formation. Overall, these studies demonstrate that human A3B is capable of driving tumor formation by a deamination-dependent mechanism and thus provide a system for studying tumor evolution and performing preclinical studies.

## Results

### A murine model for inducible expression of tumor-like levels of human A3B

To test the idea that the lack of tumor phenotypes in our original *R26-A3B* model^15^ may be due to low expression levels, we established a C57BL/6 mouse model for inducible expression of high levels of human A3B by inserting a strong *CAG* promoter upstream of the transcription stop cassette (schematics in Figure 1A; additional details in Figures S1A and S1B). Crossing *Rosa26::CAG-LSL-A3Bi* animals with CMV-Cre animals to remove the transcription stop cassette early in embryonic development results in double-transgenic progeny, here called *CAG-A3B* animals, that have human A3B protein expressed strongly in nearly all tissues including liver, pancreas, and spleen (Figure 1B). Human A3B protein levels in multiple tissues in *CAG-A3B* animals are at least 5-fold greater than levels in *R26-A3B* animals generated similarly (immunoblot in Figure 1B including quantification below). *CAG-A3B*-expressing tissues also exhibit proportionately higher ssDNA deaminase activity, with a caveat that activity in splenic extracts is challenging to quantify due to non-specific substrate cleavage by an endogenous nuclease (Figure 1C). A3B protein expression is further demonstrated by immunohistochemistry (IHC) with the rabbit monoclonal antibody 5210-87-13 (Figure 1D). Human A3B accumulates in the nuclear compartment of cells in multiple murine tissues, consistent with prior reports for human A3B subcellular localization in human cell lines and tissues.^16^^,^^17^^,^^18^^,^^19^ These observations indicate that A3B’s nuclear import mechanism is conserved, despite the fact that only distantly related polynucleotide deaminase family members are expressed in mice (i.e., Apobec3, Apobec1, and Aicda) and, importantly, that *CAG*-promoter-driven levels of human A3B are tolerated in mice without obvious phenotypes in the somatic tissues examined here.

### High A3B levels cause male-specific infertility

**Figure 2:**
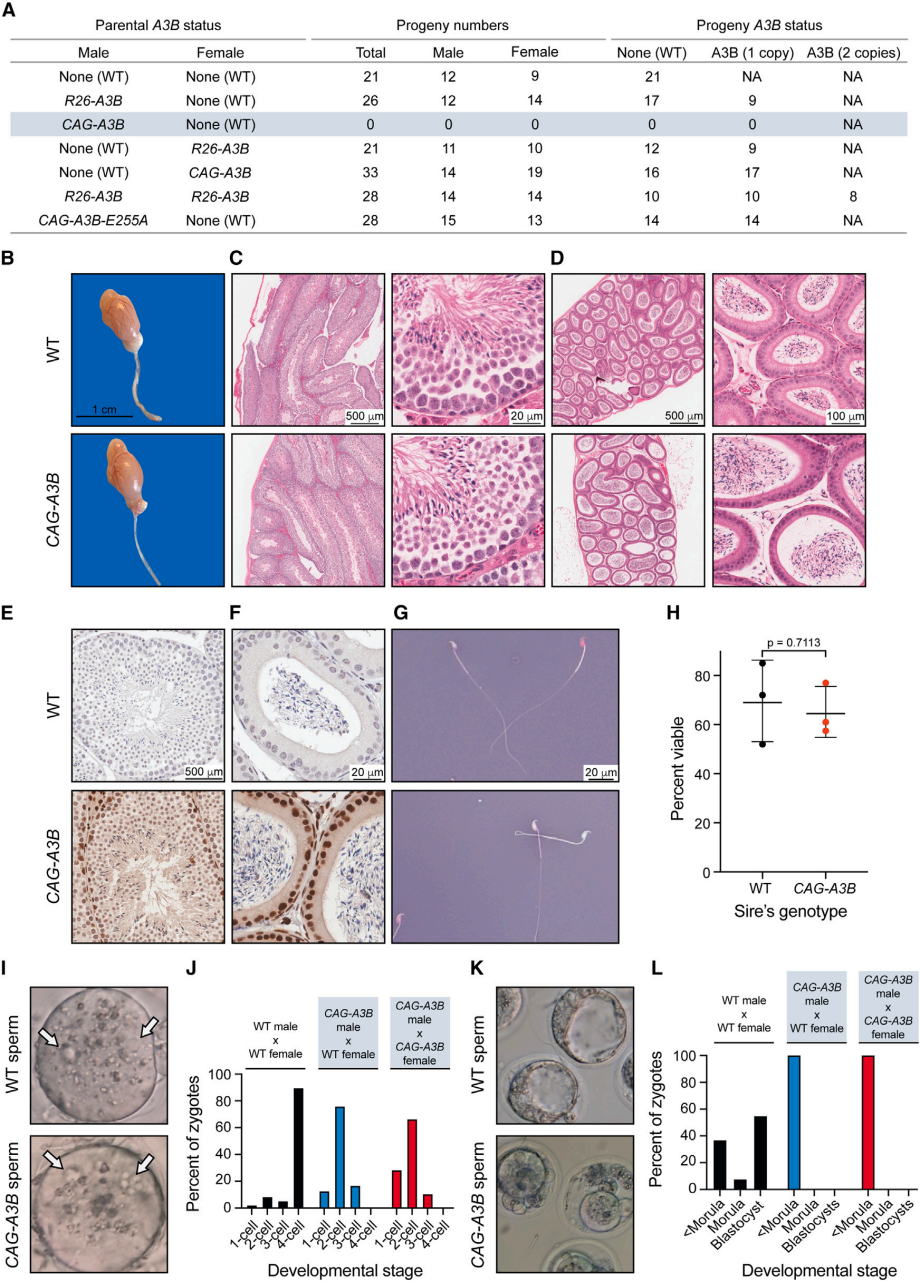
High A3B levels cause male-specific infertility (A) Progeny numbers and *A3B* status for the indicated crosses (n = 3 litters per cross). In parental animals with *A3B*, the indicated *A3B* minigene (*R26* or *CAG*) is heterozygous in combination with a WT *Rosa26* locus (data not shown). The *A3B* status of progeny is dictated by the parental cross. (B) Images of a representative testicle and epididymis from WT and *CAG-A3B* males. (C and D) H&E-stained sections of WT (top) and *CAG-A3B* (bottom) testicle and epididymis, respectively. (E and F) Anti-A3B IHC staining of the seminiferous tubule and epididymal lumen from WT and *CAG-A3B* males, respectively. (G and H) Representative images and quantification of spermatozoa from WT and *CAG-A3B* males, stained with eosin-nigrosin to distinguish live (white) and dead (pink) cells, respectively (mean ± SD of n = 200 sperm from 3 independent males; unpaired t test p value indicated). (I) Images of zygotes 7 h postfertilization of a WT ovum with spermatozoa from the indicated male genotypes. Arrows point to pronuclei, which indicate successful fertilization. (J) Proportion of embryos at the indicated developmental stage 48 h postfertilization *in vitro* (n > 50 zygotes analyzed per condition). (K) Images of developing embryos 96 h postfertilization *in vitro*. (L) Proportion of embryos at the indicated developmental stage 96 h postfertilization *in vitro* (n > 50 zygotes analyzed per condition; continuation of experiment reported in J).

During attempts to breed *CAG-A3B* animals and generate cohorts for cancer studies (as well as separate the recombined human *A3B* minigene from the CMV-Cre driver), we discovered that adult males are infertile. This phenotype is illustrated by no progeny from *CAG-A3B* male x wild-type (WT) female crosses, in comparison to near-expected Mendelian ratios from *CAG-A3B* female x WT male crosses (Figure 2A). In contrast, both male and female *R26-A3B* animals yield pups in near-Mendelian ratios. Moreover, *R26-A3B* x *R26-A3B* crosses also yield near-expected numbers of all progeny combinations, including double-homozygous animals, indicating that 2-fold more *Rosa26*-driven levels of A3B are insufficient to account for the male infertility observed with the stronger-expressing *CAG-A3B* allele.

Testes from *CAG-A3B* males appear morphologically healthy at macroscopic and microscopic levels by hematoxylin and eosin (H&E) staining (Figure 2B). Seminiferous tubules and epididymal lumen are also healthy as determined by H&E staining (Figures 2C and 2D). Moreover, high-magnification images of seminiferous tubules and epididymal lumen stain positive for human A3B and show no obvious morphological differences (Figures 2E and 2F). Notably, A3B is localized to the nuclear compartment of germ stem cells and early-stage sperm cells but seems undetectable in the late stages of sperm development, including in spermatozoa (Figure 2E). Cells within the epithelium of the epididymal lumen also express nuclear A3B, but the adjacent mature spermatozoa appear negative (Figure 2F). Moreover, mature sperm from *CAG-A3B* males appear morphologically healthy, with characteristic hook-shaped heads and functional tails of normal length (Figure 2G). Eosin-nigrosin staining also indicates no significant difference in the number of live sperm from WT versus *CAG-A3B* males (Figures 2G and 2H).

To further investigate this phenotype, WT female eggs were fertilized *in vitro* with sperm from *CAG-A3B* males and from WT males as controls. In all instances, sperm cells are able to fertilize eggs, as evidenced by the appearance of two pronuclei per ovum (Figure 2I). However, overt defects become apparent within 48 h, with all *CAG-A3B* embryos arresting before the 4-cell stage (Figure 2J). Moreover, at 96 h postfertilization, differences are even more stark with all CAG-A3B embryos visibly terminated (<morula stage development in Figures 2K–2L). In contrast, WT embryos show healthy developmental trajectories (Figures 2I–2L). These observations combine to suggest that the genetic integrity of *CAG-A3B* male sperm may be compromised. In support of this idea, as described below, this infertility phenotype requires A3B catalytic activity because male animals expressing a catalytically defective protein (E255A), which is otherwise identical to the WT enzyme, show healthy fertility (Figure 2A).

### *CAG-A3B* mice exhibit accelerated rates of tumor progression and elevated tumor numbers

**Figure 3:**
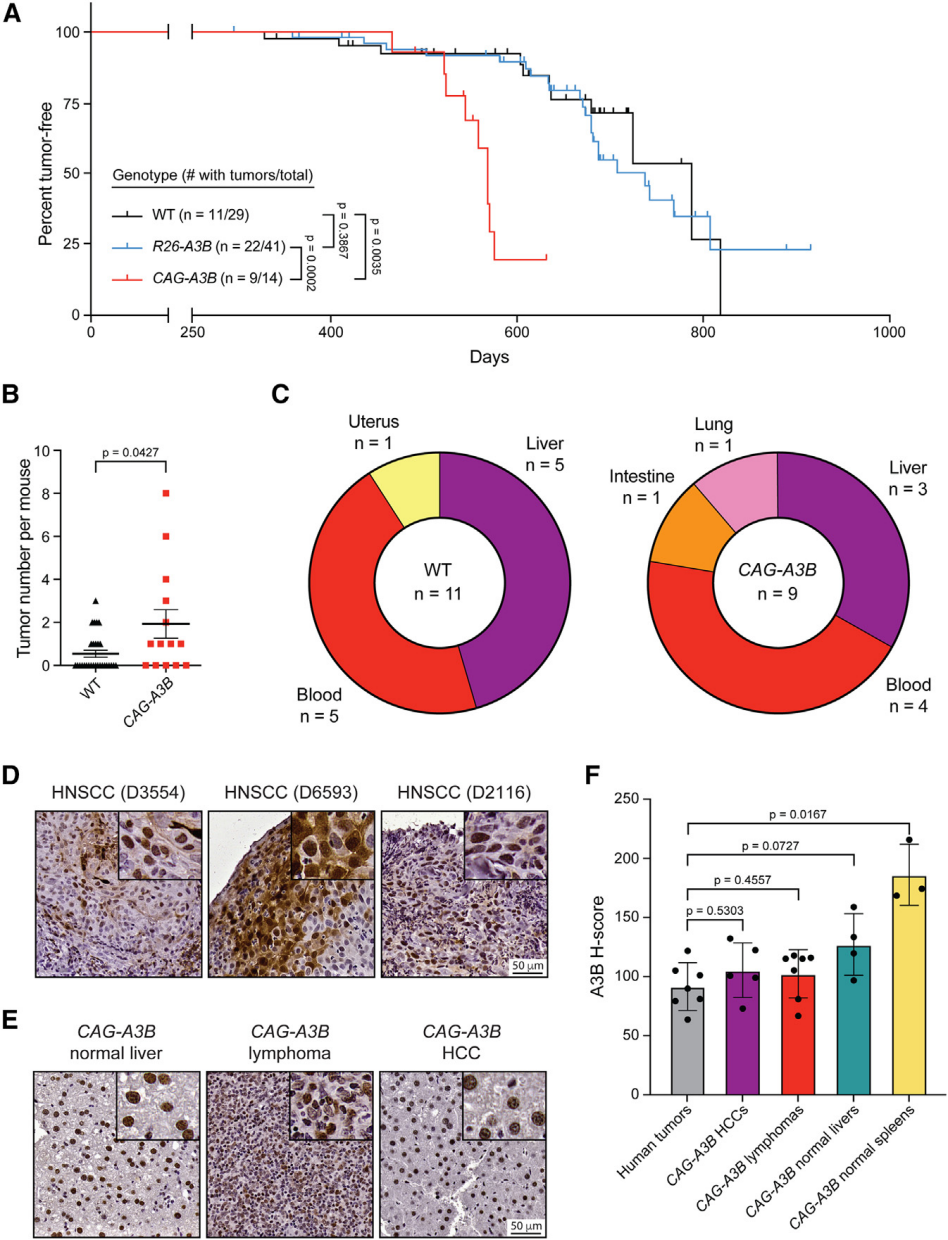
*CAG-A3B* mice exhibit accelerated rates of tumor progression and elevated tumor numbers (A) Kaplan-Meier curves comparing tumor-free survival of WT (n = 29), *R26-A3B* (n = 41), and *CAG-A3B* (n = 14) mice. The number of animals with tumors is shown over the total number of animals in each group (log rank Mantel-Cox test p values indicated). Vertical lines on each curve indicate mice that were censored. (B) Dot plot of the number of tumors per mouse in each respective genotype (mean ± SEM; Mann-Whitney U test p value indicated). (C) Pie chart summarizing primary tumor locations in WT and *CAG-A3B* mice. (D) Anti-A3B IHC staining of representative tissues from human head and neck squamous cell carcinomas (HNSCCs). Inset boxes show the same tissues at 4× additional magnification. (E) Anti-A3B IHC staining of representative tissues from *CAG-A3B* mouse tissues. Inset boxes show portions of the same tissues with 4× additional magnification. (F) Quantification of anti-A3B IHC staining in HNSCCs (n = 7), *CAG-A3B* HCCs (n = 5), *CAG-A3B* lymphomas (n = 7), *CAG-A3B* healthy liver tissues (n = 4), and *CAG-A3B* healthy spleens (n = 3) (mean ± SD; Mann-Whitney U test p values indicated). See also Figures S1–S3 and Tables S1 and S2.

Our recent studies found no difference in longevity or rates of tumor formation between WT animals and *R26-A3B* littermates expressing low levels of A3B in most tissues.^15^ This analysis is expanded here at two different animal facilities (Minneapolis and Oslo), and again, no substantial difference in mouse development or overall rates of tumor formation is observed (Figures 3A and S1C). However, a slight increase in lymphoma frequency may be apparent in the Oslo facility, where animals are housed in a minimal disease unit (Figure S1D), but this modest phenotype is not accompanied by elevated mutation loads or an obvious APOBEC3 mutation signature (Figures S1E and S1F). An independent model, in which human *A3B* (as a *turbo-GFP* fusion) is integrated at the *Col1a1* locus and expressed inducibly using a *R26*-integrated tetracycline transactivator,^20^ also yields modest A3B expression levels and no significant tumor phenotypes (Figure S2).

In contrast to these models that directly or indirectly express low levels of human A3B, the *CAG-A3B* model with full-body A3B expression shows accelerated rates of tumor formation (Figure 3A). By 600 days, over 50% of *CAG-A3B* animals have developed tumors, whereas less than 20% of WT mice are penetrant at this time point (Figure 3A; summary of all *CAG-A3B* tumor information in Table S1). *CAG-A3B* animals also have significantly higher tumor burdens as compared with WT mice, consistent with accelerated levels of mutagenesis (Figure 3B). Most of the tumors in *CAG-A3B* animals are lymphomas or hepatocellular carcinomas (HCCs) (Figure 3C). WT animals also show a similar spectrum of tumors (albeit with longer latencies), suggesting that A3B may accelerate the penetrance of preexisting cancer predispositions (Figure 3C). In support of this possibility, MMTV-Cre is known to have leaky expression in hematopoietic cells,^21^^,^^22^^,^^23^ and, accordingly, attempts to induce *CAG-A3B* specifically in mammary epithelial cells also trigger the formation of lymphomas (Figure S3). Importantly, levels of human A3B protein in the nuclei of murine tumor cells approximate the upper amounts observed in the nuclei of human tumor cells (e.g., IHC for representative head and neck squamous cell carcinomas in Figure 3D, with comparison to additional murine tumor and healthy tissues in Figure 3E and quantification in Figure 3F; additional head and neck squamous cell carcinoma [HNSCC] information in Table S2).

### Heterogeneity and evidence for metastasis in tumors from *CAG-A3B* animals

**Figure 4:**
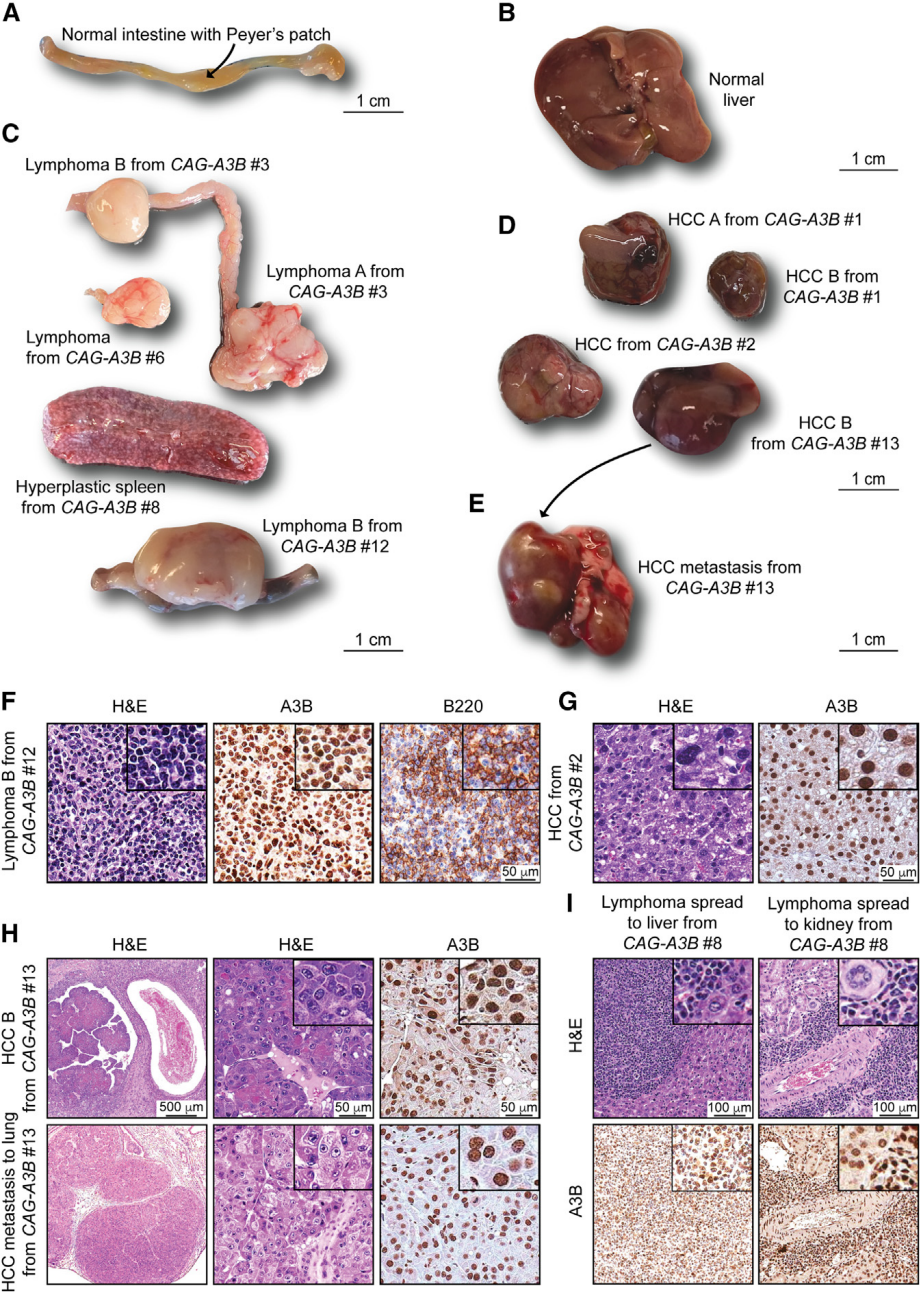
Heterogeneity and evidence for metastasis in tumors from *CAG-A3B* animals (A and B) Representative healthy intestine with Peyer’s patch (arrow) and healthy liver tissues, respectively, from *CAG-A3B* mice. (C and D) Macroscopic pictures of a heterogeneous assortment of lymphomas and HCCs, respectively, from *CAG-A3B* mice. (E) Representative image of a primary HCC that metastasized to the lung (HCC B from *CAG-A3B* #13 in D). (F) H&E, anti-A3B, and anti-B220 IHC of lymphoma B from *CAG-A3B* #12. Inset boxes show portions of the same tumors at 4× additional magnification. (G) H&E and anti-A3B IHC of HCC from *CAG-A3B* #2. Inset boxes show portions of the same tumors at 4× additional magnification. (H) H&E and anti-A3B IHC staining of a primary HCC (top) and its metastatic dissemination to the lung (bottom) from *CAG-A3B* #13. Inset boxes show portions of the same tumors at 4× additional magnification. (I) H&E and anti-A3B IHC staining of a diffuse large B cell lymphoma in the liver (left) and kidney (right). Inset boxes show the same tumors at 4× additional magnification. See also Figure S4 and Table S1.

Tumors that develop in *CAG-A3B* animals are visibly heterogeneous, which is a hallmark of human tumor pathology that has been difficult to recapitulate in mice (Figures 4A–4D). For instance, in comparison to healthy intestine-associated lymphoid follicles in Figure 4A and a healthy liver in Figure 4B, both lymphomas and HCCs show significant visible heterogeneity (pictures of representative tumors in Figures 4C and 4D; additional *CAG-A3B* tumor information in Table S1). Both of these tumor types are variable for a range of characteristics including size, morphology, color, and vascularization. For example, HCC B from *CAG-A3B* #1 and HCC from *CAG-A3B* #2 from independent animals show differential morphology, colorization, and vasculature.

We next characterized tumors at the cellular level by H&E and IHC for select diagnostic markers. First, all tumors showed diffuse, strong, nuclear-only A3B staining in the entirety of the lesional cells (Figures 4F–4I, S4A, and S4B). Most lymphomas appear to be comprised of a uniform proliferation of atypical lymphoid cells with round or ovoid hyperchromatic nuclei showing marked nuclear pleomorphism, increased numbers of mitotic figures, and scant eosinophilic cytoplasm (Figures 4F and S4A). A fraction of lesions also appear macroscopically as enlarged spleens with features suggestive of splenic lymphoid hyperplasia and variable increases in the number and size of follicular structures (Figures 4C and S4B). Second, staining with the diagnostic B cell marker B220 indicates that the *CAG-A3B* mice are developing predominantly B cell lymphomas, either *de novo* or from preceding lymphoid hyperplasias (Figures 4F, S4A, and S4B). This inference is supported by the clonality of antibody gene contigs derived from RNA sequencing (RNA-seq) data, indicating that tumorigenesis occurs after V(D)J recombination in the B cell lineage (Figure S4C). However, several B cell lymphomas are accompanied by abnormally large, mostly non-clonal, T cell populations, as determined by CD3 staining, *Thy-1* mRNA levels, and diverse T cell receptor (TCR) junctions, which may be the result of strong anti-tumor T cell responses and/or inflammation in the tumor microenvironment (Figures S4A–S4D). Levels of the DNA damage marker γ-H2AX also trend higher in *CAG-A3B* HCCs in comparison with WT HCCs (Figures S4E and S4F). However, in contrast to near-uniform A3B staining, only a subset of cells are positive for γ-H2AX staining, suggesting the involvement of other factors such as cell-cycle stage.

Importantly, a subset of *CAG-A3B* animals also show evidence of distant organ metastasis, or disseminated lymphoproliferative malignancy, with one case of HCC metastasizing to the lung, one case of disseminated lymphoma involving Peyer’s patches and intestinal mucosa, two cases of lymphoma with diffuse lymph node dissemination to multiple lymph nodes, and one case of lymphoma spreading to multiple lymph nodes, the liver, and the kidney (e.g., Figures 4C, 4E, 4H, and 4I). In several instances, both the primary and the metastatic lesions are located adjacent to blood vessels (Figures 4H and 4I). In the case of liver-to-lung metastasis, the metastatic tumor shows indistinguishable histopathologic features from the primary HCC and similarly uniform and strong A3B positivity (Figure 4H). In agreement with this, all disseminated lymphoproliferative lesions also show strong A3B nuclear-only immunostaining (Figures 4I, S4A, and S4B), which differs from our prior studies in which human A3A protein expression is selected against and disappears in early stages of HCC development.^13^ No metastases were observed in the WT mice over the same time frame. These observations combine to suggest that A3B influences both early- and late-stage tumor development in an ongoing manner.

### *CAG-A3B* tumors exhibit an APOBEC3 mutation signature

**Figure 5:**
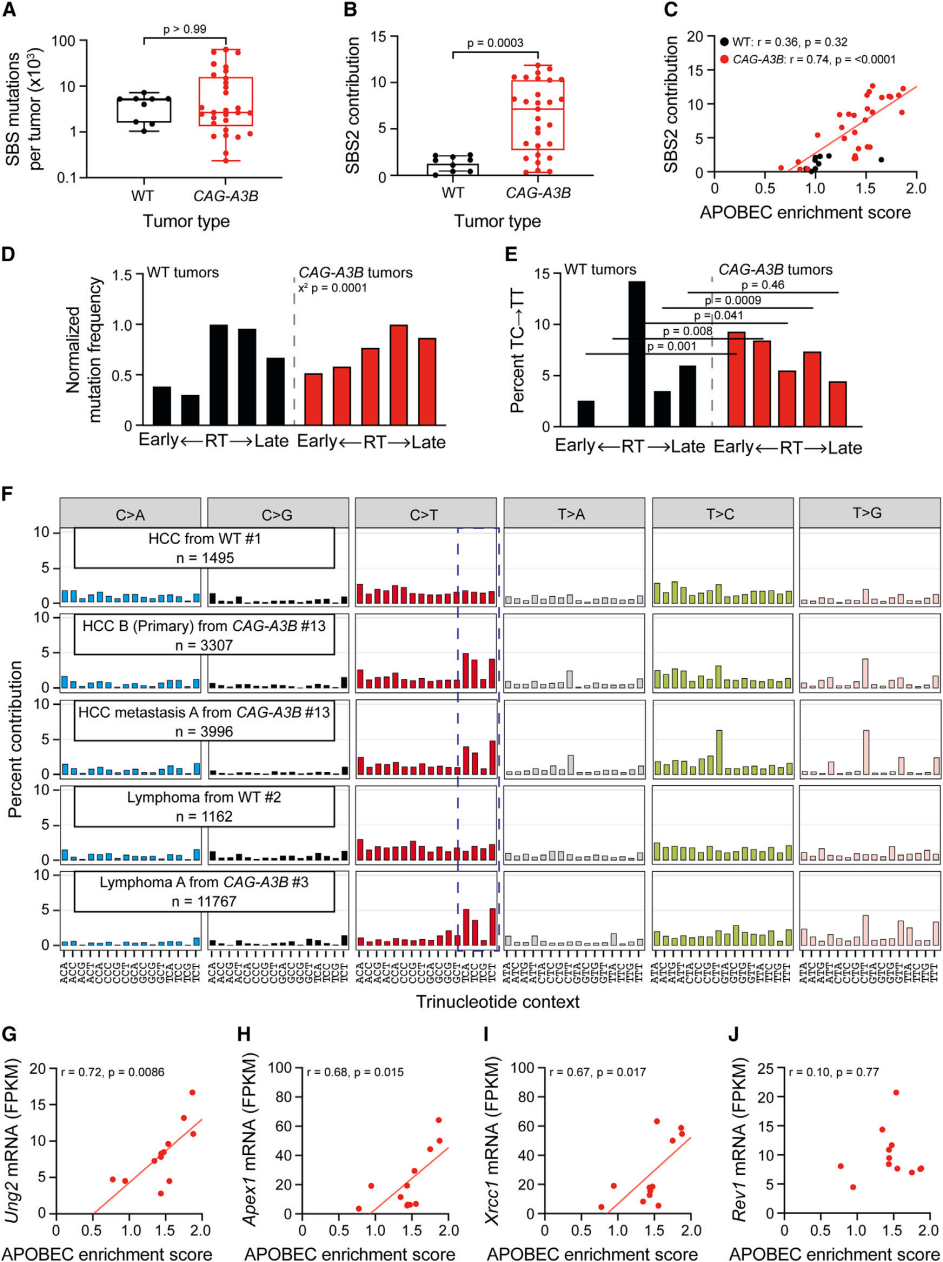
*CAG-A3B* tumors exhibit APOBEC3 signature mutations (A and B) Box and whisker plots of the total number of SBS mutations and the percentage of SBS2, respectively, in tumors from WT and *CAG-A3B* mice. The middle horizontal line is the median, the top and bottom of the box specify the upper and lower quartiles, and the whiskers outside the box represent the maximum and minimum values (Mann-Whitney U test p value indicated). (C) Scatterplots comparing APOBEC mutation signature enrichment scores to the percentage contribution of SBS2 in tumors from WT and *CAG-A3B* animals (Pearson correlation coefficient and p values indicated). Linear regression shown for *CAG-A3B* data (not possible for WT). (D) Bar plots showing the proportion of mutations in WT and *CAG-A3B* tumors according to early- to late-replicating regions (mutation numbers normalized to the largest quintile in each group). The chi-squared test p value is indicated. (E) Bar plots showing the percentage of TC-to-TT mutations as a percentage of all mutations in each quintile in (D) (Mann-Whitney U-test p values indicated). (F) Representative SBS mutation profiles for the indicated tumors from WT or *CAG-A3B* animals (mutation numbers shown). The dashed box highlights APOBEC3-preferred TC motifs characteristic of SBS2. (G–J) Scatterplots of APOBEC enrichment scores from *CAG-A3B* lymphomas (n = 12) compared to the mRNA levels of *Ung2*, *Apex1*, *Xrcc1*, and *Rev1*, respectively, from the same tumors (linear regression lines and Pearson correlation coefficients and corresponding p values indicated). See also Figures S5–S7 and Table S1.

Whole-genome sequencing (WGS) of tumors from *CAG-A3B* and WT animals, in comparison to matched tail DNA, enables somatic mutation landscapes to be compared and underlying mutational processes deduced. Tumors from both *CAG-A3B* and WT animals exhibit large numbers of all types of SBS mutations (*CAG-A3B*: n = 29 tumors, SBS range = 344–62,496, mean = 11,791, median = 2,659; WT: n = 9 tumors, SBS range = 1,047–7,242, mean = 4,051, median = 5,221; Figure 5A). Although mean SBS numbers are statistically indistinguishable in tumors from WT and *CAG-A3B* mice, larger proportions of C-to-T mutations in TCA, TCC, and TCT trinucleotide motifs (SBS2) are evident in tumors from *CAG-A3B* animals (Figures 5A and 5B). As expected, the percentage of SBS2 mutations in tumors from *CAG-A3B* mice associates positively with the overall APOBEC mutation signature enrichment score (red data points and linear regression in Figure 5C). In comparison, tumors from WT animals do not show such an association (black data points in Figure 5C). The vast majority of A3B-associated C-to-T mutations are dispersed and do not occur in small or large clusters of APOBEC3 signature events called *omikli* and *kataegis*, respectively.^24^^,^^25^^,^^26^^,^^27^^,^^28^ However, in line with prior reports,^29^^,^^30^ *CAG-A3B* tumors also show a mutational bias in genomic regions associated with early DNA replication timing (Figure 5D). This early replication bias becomes even more pronounced when only TC-to-TT SBS2 mutations are considered (Figure 5E).

Trinucleotide SBS mutation distributions are shown for representative WT and *CAG-A3B* tumors in Figure 5F. As alluded to above, only tumors from *CAG-A3B* animals show elevated percentages of SBS2 mutations in TCA, TCC, and TCT trinucleotide motifs (e.g., HCC B from *CAG-A3B* #13 and lymphoma A from *CAG-A3B* #3 in Figure 5F). Curiously, C-to-G and C-to-A transversion mutations in the same TC-focused motifs (SBS13) are not apparent above background levels in the same tumors. These two mutation signatures, SBS2 and SBS13, are thought to be alternative mutational outcomes of APOBEC3-catalyzed C-to-U deamination events, with the latter signature attributable to uracil excision by uracil DNA glycosylase 2 (Ung2), followed by a C insertion opposite the newly created abasic site by the DNA polymerase Rev1 (and/or another translesion-synthesis DNA polymerase). Interestingly, RNA-seq data from *CAG-A3B* lymphomas indicate positive associations between APOBEC mutation signature enrichment scores and mRNA levels for *Ung2*, *AP endonuclease 1* (*Apex1*), and *X-ray repair cross-complementing protein 1* (*Xrcc1*) but not for *Rev1* (Figures 5G–5J). Conversely, lymphomas from WT animals lack an association between APOBEC enrichment scores and these transcripts and, in the case of *Apex1*, may exhibit a negative association (Figures S5A–S5D). Thus, the absence of SBS13 in *CAG-A3B* tumors may be explained by elevated rates of error-free repair (fewer persisting abasic sites) and/or insufficient Rev1 (leaving more opportunities for DNA polymerases that follow the A rule to misincorporate dAMP opposite an abasic site).

A *de novo* extraction of the SBS mutation signatures in the entire set of murine tumors yields five distinct signatures, including one closely resembling SBS2 (SigA in Figures S5E and S5F). Over half of the tumors from *CAG-A3B* animals show >10% SigA, whereas all of the tumors from WT animals have <10% (Figure S5E). SigB and SigC resemble SBS5 (unknown process associated with aging) and SBS17 (potentially oxidative damage), as defined by analyses of human cancers^4^^,^^5^^,^^31^ and also reported in mice.^32^^,^^33^^,^^34^ SigD and SigE resemble SBS9 and SBS28, and, interestingly, these two signatures appear over-represented in *CAG-A3B* tumors (Figure S5E). The molecular basis for the SigD bias is unclear at present, but the SigE bias may be due to a single amino acid substitution, Lys70Thr, in DNA polymerase κ (6/6 tumors from the same animal have an A209-to-C mutation in *Polk*).

WGS of multiple tumors from unrelated animals also unambiguously demonstrates the clonal relationship between primary tumors and inferred metastatic outgrowths (inferred above in immunohistological analyses). For instance, HCC B and the associated metastasis to the lung in *CAG-A3B* animal #13 share several morphological features as described above in Figure 4H, and WGS shows a total of 344 common base substitution mutations and over 3,000 private mutations in this metastasis, consistent with ongoing mutagenesis and tumor evolution contributing to the observed metastatic phenotype (Figure 5F). Hierarchical clustering (PyClone-VI)^35^ analysis also indicates that tumors from *CAG-A3B* animals with high APOBEC mutation signature enrichment scores (ES^high^) exhibit greater intralesion subclonal diversity in comparison with tumors from *CAG-A3B* animals with low APOBEC mutation signature enrichment scores (ES^low^), consistent with ongoing tumor evolution catalyzed by A3B (Figures S6A and S6B). Intralesion subclonal diversity is also high in tumors from WT animals, but this does not involve A3B and may be attributable in part to the longer durations required for tumor formation.

### DNA deaminase activity is required for A3B-driven tumor phenotypes

**Figure 6:**
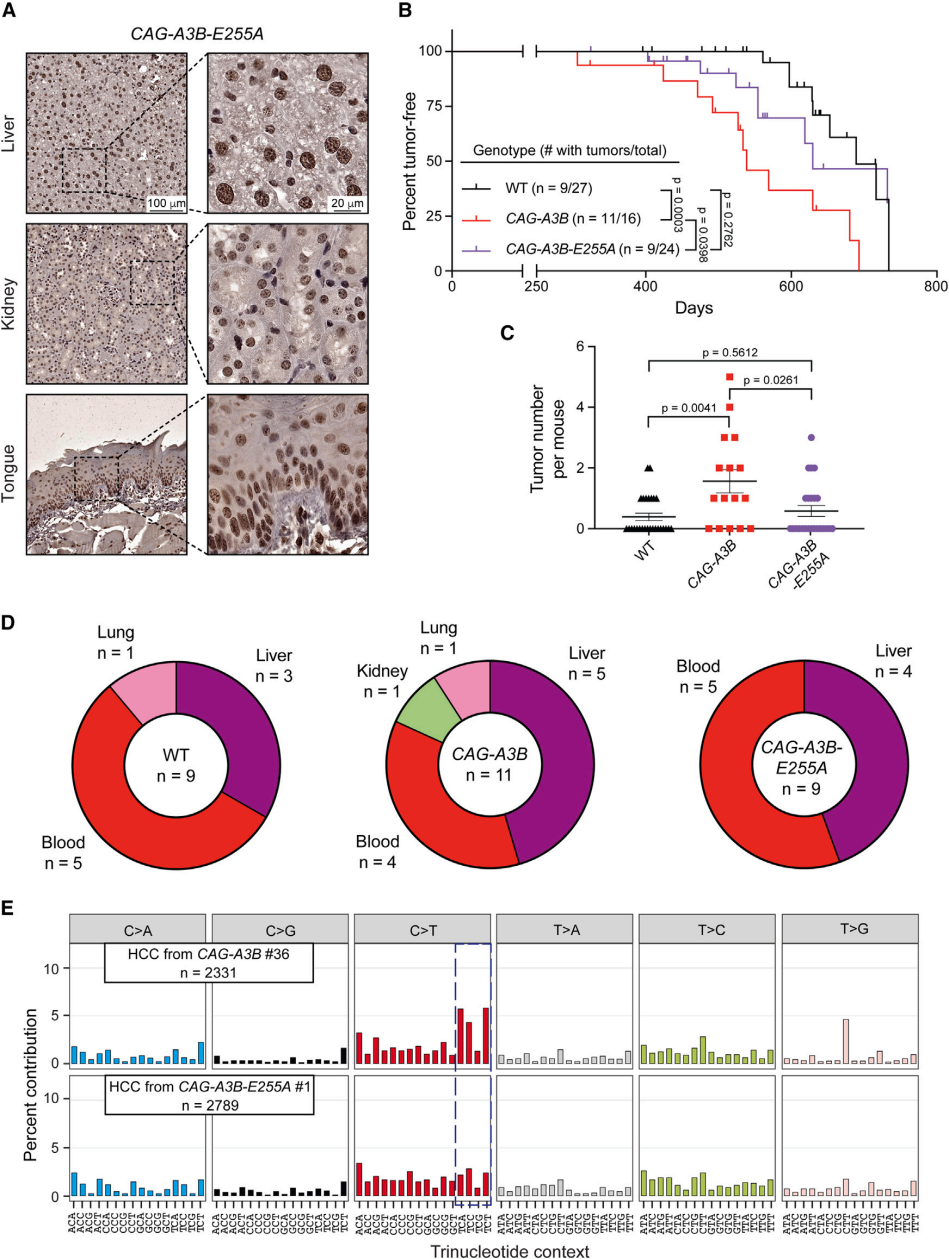
DNA deaminase activity is required for A3B-driven tumor phenotypes (A) Anti-A3B IHC staining of representative tissues from *CAG-A3B-E255A* mice (40× magnifications on right are enlargements of regions of the corresponding 10× images on left). (B) Kaplan-Meier curves comparing tumor-free survival of WT (n = 27), *CAG-A3B* (n = 16), and *CAG-A3B-E255A* (n = 24) mice (log rank Mantel-Cox test p values indicated). The number of animals with tumors is shown over the total number of animals in each group. Vertical lines on each curve indicate mice that were censored. (C) Dot plot of the number of tumors per mouse in each respective genotype (mean ± SEM; Mann-Whitney U test p values indicated). (D) Pie chart summarizing tumor locations in *CAG-A3B* and *CAG-A3B-E255A* mice. (E) Representative SBS mutation profiles for the indicated tumors from *CAG-A3B* and *CAG-A3B-E255A* animals (mutation numbers shown). The dashed box highlights APOBEC3-preferred TC motifs characteristic of SBS2. See also Figure S6.

To formally test whether the phenotypes described above are due to deaminase activity, we generated an additional knockin capable of inducibly expressing a well-characterized catalytic mutant of A3B with glutamic acid 255 mutated to alanine^36^^,^^37^^,^^38^ (*Rosa26::CAG-LSL-A3Bi-E255A*). These animals were crossed with CMV-Cre mice to remove the transcriptional stop cassette and generate progeny with whole-body expression of A3B-E255A (i.e., *CAG-A3B-E255A*). Males and females are fertile with near-expected Mendelian progeny ratios, in contrast to the male sterility described above for *CAG-A3B* males (Figure 2). The A3B-E255A protein appears predominantly nuclear by IHC and indistinguishable in appearance from the WT enzyme (Figure 6A).

Importantly, longer-term studies of *CAG-A3B-E255A* animals yielded a Kaplan-Meier plot and tumor burdens similar to that of WT C57BL/6 animals (Figures 6B and 6C). In contrast, but similar to results described above in Figure 3, *CAG-A3B* animals exhibit accelerated rates of tumor formation and higher numbers of tumors per animals (Figures 6B and 6C). HCCs and lymphomas are the predominant tumor types in WT animals, *CAG-A3B* animals, and *CAG-A3B-E255A* animals (Figure 6D, compare with Figure 3C). As expected, WGS of representative tumors from *CAG-A3B* and *CAG-A3B-E255A* animals only yields an APOBEC3 mutation signature (SBS2) in the former group (Figure 6E). In comparison, RNA editing profiles extracted from *CAG-A3B* and WT tumor RNA-seq datasets are similar, with no noticeable edits attributable to A3B (Figure S6C). These results are consistent with a DNA-deamination-dependent mechanism being responsible for both the observed male sterility and the accelerated tumor formation phenotypes of *CAG-A3B* animals.

### *CAG-A3B* tumors exhibit increased structural variation

**Figure 7:**
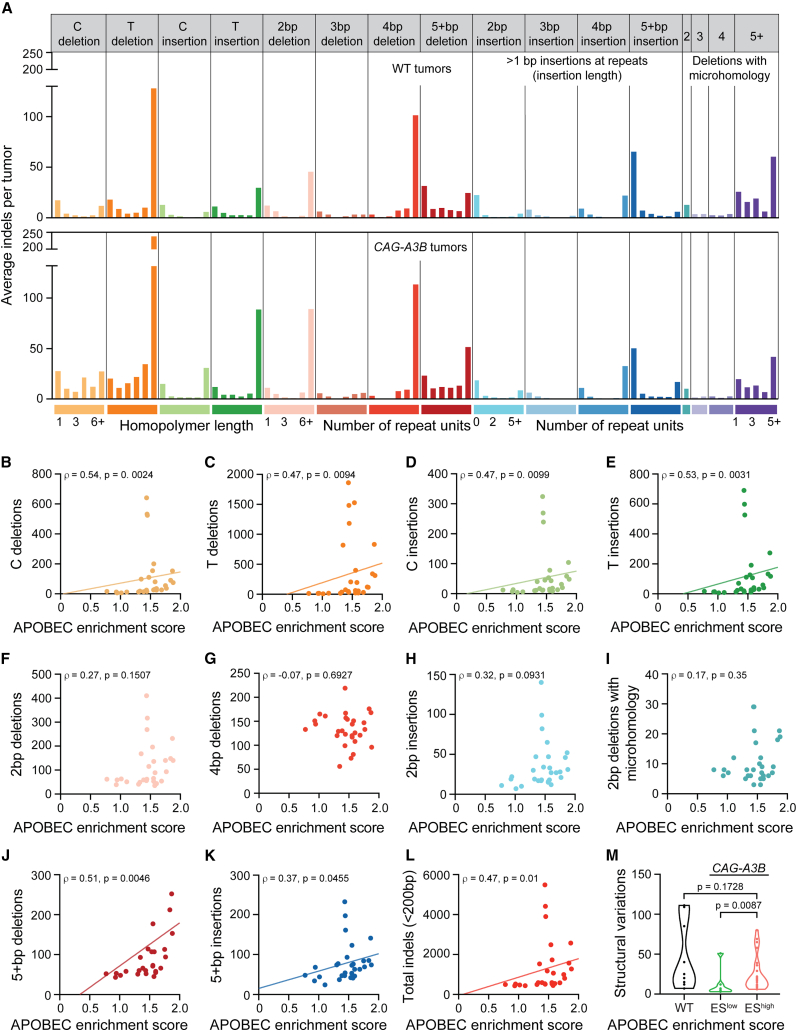
Hypermutated *CAG-A3B* tumors also exhibit higher frequencies of a range of structural variations (A) Composite spectrum of the average number of small indels in tumors from WT (n = 9) and *CAG-A3B* (n = 29) animals. (B–K) Scatterplots showing relationships between APOBEC mutation signature enrichment scores from *CAG-A3B* tumors and the indicated indel types (linear regression lines, except F–I, and Spearman’s rank correlation coefficients and corresponding p values indicated). (L) Scatterplot showing the relationship between APOBEC mutation signature enrichment scores from *CAG-A3B* tumors and the total number of indels <200 bp in each tumor (linear regression line and Spearman’s rank correlation coefficient and corresponding p value indicated). (M) Violin plots of the total number of structural variations in tumors from WT mice in comparison with tumors from *CAG-A3B* animals with low or high APOBEC mutation signature enrichment scores (ES; Mann-Whitney U test p values indicated). See also Figure S7.

Given prior reports,^39^^,^^40^^,^^41^ we are also interested in determining whether A3B causes structural variation. A subset of A3B-catalyzed deamination events may become abasic sites, ssDNA nicks, and double-stranded (ds)DNA breaks and be processed into a wide variety of different non-SBS mutagenic outcomes. We first quantified small-scale events ranging from single-nucleotide indel mutations to larger-scale indels <200 bp (Figure 7A). Interestingly, the intensity of the APOBEC mutation signature (enrichment score) associates positively with single T/A indels and single C/G indels in tumors from *CAG-A3B* animals but not in those from control WT animals (Figures 7B–7E and S7A–S7D, respectively). In comparison, no significant differences are seen with 2, 3, and 4 bp indels or with 2, 3, and 4 deletions with microhomology, which might reflect the relatively small number of events in each of these categories (Figure 7A, 7F–7I, and S7E–S7H). Accordingly, APOBEC mutation signature enrichment scores do correlate with indels in the larger 5+ category (5 to 200 bp) in *CAG-A3B* tumors (Figures 7J and 7K) but not in control tumors from WT animals (Figures S7I–S7J). As anticipated from these results, the total sum of all indel events in *CAG-A3B* tumors also associates positively with the APOBEC mutation signature enrichment scores, consistent with both of these mutational outcomes sharing A3B-dependent DNA deamination as a common mechanistic origin (Figure 7L and WT data for comparison in Figure S7K).

One must also consider larger structural variations including indels >500 bp, inversions, translocations, and more complex events. We therefore quantified these structural variations in tumors from WT mice and in tumors from *CAG-A3B* mice with ES^low^ and ES^high^. Interestingly, a statistically higher level of structural variation is evident in tumors with ES^high^ in comparison with those with ES^low^ (Figure 7M). Tumors from WT mice can also exhibit high numbers of structural variation, which may be due in part to the fact that these animals are necessarily older (Figure 7M). Indeed, a linear correlation exists between structural variation level and the age of the WT animals from which the tumor originated (Figure S7L). Similar positive correlations with WT animal age are apparent for net levels of SBS mutations and indels (Figures S7M and S7N). These results are consistent with an age-related mutational mechanism producing the genomic alterations that predominate in WT mice and that can be eclipsed by A3B-driven events in *CAG-A3B* animals.

## Discussion

The studies here demonstrate that human A3B is capable of driving tumor formation *in vivo* by accelerating rates of primary tumor development as well as by triggering secondary growths (i.e., metastases). Specifically, full-body expression of *CAG*-promoter-driven levels of human A3B, which approximate those in human tumors, results in accelerated rates of B cell lymphomagenesis and hepatocellular carcinogenesis as well as a smaller number of other tumor types. Nearly all tumors in A3B-expressing animals exhibit elevated levels of C-to-T mutations in TC dinucleotide motifs (SBS2) consistent with the established biochemical activity of this ssDNA deaminase. Catalytic activity is required for all A3B-associated phenotypes, as evidenced by healthy fertility and long-term analyses of otherwise isogenic *CAG-A3B-E255A* animals. APOBEC mutation signature enrichments also associate positively with macroscopic and subclonal tumor heterogeneity, as well as with multiple types of indel mutations. A significantly elevated level of structural variation is also apparent in *CAG-A3B* tumors with ES^high^ in comparison with those with ES^low^. Altogether, these observations support a continuous tumor evolution model in which a subset of A3B-catalyzed C-to-U DNA deamination events lead to signature C-to-T mutations and other deamination events are processed by uracil base excision repair enzymes into ssDNA breaks that can be converted into indels and larger-scale chromosome aberrations.^9^^,^^42^^,^^43^

Our studies also led to two major unexpected results. First, only C-to-T mutations characteristic of SBS2 are evident in A3B-expressing murine tumors and not C-to-G mutations characteristic of SBS13. These two mutation signatures are frequently coincident in human tumors but can occur separately as reported for over 20 B cell lymphoma cell lines, urothelial carcinomas with micropapillary histology, Apc^Min^ colorectal tumors in A3A transgenic mice, and yeast and human cells defective in uracil DNA glycosylase or the translesion DNA polymerase Rev1.^11^^,^^13^^,^^44^^,^^45^^,^^46^^,^^47^ A possible molecular mechanism is suggested by low *Rev1* expression levels in A3B-expressing tumors here and a lack of an association between these levels and APOBEC mutation signature enrichment. If Rev1 is not present to insert C opposite abasic sites (downstream of A3B deamination of C and Ung2 excision of the resulting U), then another DNA polymerase that follows the A-insertion rule is likely to substitute and contribute to the observed C-to-T transition mutation bias. Alternatively, the C-to-T mutation bias may be due simply to lower Ung activity in murine tissues as compared to humans,^48^ though this explanation is difficult to reconcile with the fact that transient expression of A3A can lead to both SBS2 and SBS13 in a murine model for HCC.^13^

Second, A3B-expressing males (but not females) are completely sterile, and this phenotype is also completely dependent on catalytic activity. Our studies indicate that testes and sperm are morphologically healthy and that the defect manifests postfertilization between the 2- and 4-cell stages of development. This result is consistent with the genetic instability reported above, and additional studies will be needed to delineate the precise defect(s). This result contrasts with most male-specific infertilities, which manifest in part by underdeveloped testes.^49^^,^^50^^,^^51^ It is additionally curious that males are affected specifically and that whole-body A3B-expressing females have thus far been fertile for over 20 generations.

The results here with the full-body *CAG-A3B* model represent a major advance over our prior work with a full-body *R26-A3B* model (Boumelha et al.^15^ and expanded on here). The key difference between these models is higher but still human tumor-like levels of *A3B* expression from the stronger *CAG* promoter. Most importantly, the *CAG-A3B* model enabled us to demonstrate that A3B alone is capable of driving tumor formation through a mechanism that requires catalytic activity and results in APOBEC3 signature mutations as well as associated indels and larger-scale structural variations. However, many questions remain unresolved, including what combination of genetic (and perhaps even epigenetic) events caused by A3B are required for primary tumor formation and metastasis development. Much larger numbers of tumor genome sequences will be required to answer this question and identify potential driver mutations. For instance, based on a human cancer gene list,^52^ individual *CAG-A3B* tumors reported here have APOBEC3 signature mutations in several established cancer genes including *Apc* (Glu2182Lys) and *Plcg1* (Glu1163Lys), as well as APOBEC3-associated events in other cancer genes such as a frameshift mutation in a TC motif in *Nf2* (Glu541fs). Surprisingly, despite multiple studies associating p53 loss of function in A3B mutagenesis,^42^^,^^53^^,^^54^^,^^55^ no mutations or copy-number variations are evident thus far in *Trp53* in *CAG-A3B* tumor genomes.

It is also important to recognize that the *CAG-A3B* model described here has some limitations. First, A3B expression is constitutively driven from a *CAG* promoter, which contrasts with the regulation of endogenous A3B in humans with peak protein levels reported in cell lines and tumors at the G2/M phase of the cell cycle.^56^^,^^57^ The constitutive nature of the *CAG* promoter also makes it tricky to dissociate tumor-cell-autonomous from -non-autonomous roles for A3B in tumor formation. Second, *CAG-A3B* animals express A3B protein levels similar to those observed at the high end of cells within human tumors (Figures 3D–3F). Therefore, it is difficult to study the impact of lower expression levels, although our original *R26-A3B* model^15^ may suffice for that purpose (e.g., Mayekar et al.^58^). Third, we have yet to combine the *CAG-A3B* minigene in a tissue-specific manner to address potential interactions with known drivers such as signal transduction activation. For instance, initial studies with *R26-A3B* in an EGFR-driven lung cancer model indicate that A3B is capable of fueling tumor evolution and contributing to drug resistance (even in the absence of inflicting overt APOBEC3 signature mutations).^58^ Last, once *CAG-A3B* expression is activated by Cre-mediated removal of the transcription stop cassette, it cannot be easily turned off. Thus, additional modifications to this model will be necessary, for instance, to address whether continued tumor development and metastasis might require ongoing A3B mutational activity.

Since the first implication of A3B in cancer,^25^^,^^26^^,^^42^^,^^59^ there has been an urgent need to develop a robust mouse model for mechanistic and preclinical studies. Our first attempt resulted in transgene inactivation, most likely by A3B selecting against itself and promoting its own inactivation.^13^ Our second attempt used the endogenous *Rosa26* promoter to drive human *A3B* expression.^15^^,^^58^ This model enables modest A3B expression levels in most murine tissues, healthy fertility, no overt cancer phenotypes, and subtle effects in lung tumor models in the presence of drug selection (without APOBEC3 signature mutations).^15^ Our third attempt, reported here, leads to higher, human tumor-like levels of A3B in most murine tissues. These “just right” or “Goldilocks” levels of human A3B accelerate rates of tumorigenesis associated with an accumulation of APOBEC3 signature mutations, a variety of different indels, and larger-scale chromosomal aberrations. Therefore, together with the Cre-inducibility of the system, the *CAG-A3B* and *CAG-A3B-E255A* models described here may be helpful for directing tissue-specific expression of this DNA-mutating enzyme and studying additional human cancer types that show high frequencies of APOBEC3 signature mutations including those of the bladder, breast, cervix, head/neck, and lung and of other tissue types.

## Limitations of the study

The *CAG-A3B* model described here expresses human *A3B* constitutively from a heterologous *CAG* promoter. This separates expression of this gene from regulatory mechanisms that are normally operative in human cells including transcriptional repression and cell-cycle regulation (although some of the same regulatory mechanisms are often dysregulated in human tumors).^56^^,^^57^ This study does did not experimentally address alternative mechanisms such as roles for A3B in other (non-tumor) cell types, roles for A3B in altering the epigenome (e.g., methyl-C landscape),^60^ or roles for A3B in transcriptional regulation^53^ or R-loop homeostasis.^37^

## STAR★Methods

### Key resources table

**Table:** 

REAGENT or RESOURCE	SOURCE	IDENTIFIER
**Antibodies**		

Rabbit monoclonal anti-CD3	Abcam	Cat# ab16669 RRID: AB_443425
Rabbit monoclonal anti-A3B	Brown et al.^17^	5210-87-13; RRID: AB_2721202
Rat monoclonal anti-B220	BD Pharmogen	Cat# 550286 RRID: AB_393581
Rabbit monoclonal anti-γ-H2AX	Cell Signaling	Cat# 9718 RRID: AB_2118009
Mouse monoclonal anti-tubulin	Sigma-Aldrich	Cat# T5168 RRID: AB_477579
Rabbit anti-actin	Sigma-Aldrich	Cat# A2066 RRID: AB_476693
Goat anti-rabbit HRP	Cell Signaling	Cat# 7074P2 RRID: AB_2099233
Goat anti-mouse IRdye 800CW	LI-COR	Cat# 926-32210 RRID: AB_621842

**Biological samples**		

Human Head and Neck FFPE sections	Argyris et al.^61^	Table S2

**Chemicals, peptides, and recombinant proteins**		

Cytoseal	Thermo Fisher Scientific	Cat# 23-244257
CitriSolv	Decon Labs	Cat# 1601
Dulbecco’s Modified Eagle Medium (DMEM)	Gibco	Cat# 11965-084
Fetal Bovine Serum (FBS)	Gibco	Cat# A52567-01
Reveal decloaker	Biocare	Cat# RV1000M
Background sniper	Biocare	Cat# BS966
TransIT-LT1 Transfection Reagent	Mirus	Cat# MIR2304
Penicillin-Streptomycin	Gibco	Cat# 15140122
MEM, GlutaMAX™ Supplement	Gibco	Cat# 41090036
MEM Non-Essential Amino Acids Solution	Gibco	Cat# 11140050
2-Mercaptoethanol	Gibco	Cat# 21985023
Novolink Max Polymer detection system	Leica Biosystems	Cat# RE7280-CE
Mayer’s Hematoxylin	Electron Microscopy Sciences	Cat# 26043-06
Permount	Thermo Fisher Scientific	Cat# SP15-100
cOmplete protease inhibitor	Sigma-Aldrich	Cat# 11697498001
RNase A	Qiagen	Cat# 19101
Poly-Prep chromatography columns	Bio-Rad	Cat# 7311550
Ni-NTA Superflow	Qiagen	Cat# 30410
Doxycycline rodent diet (625 mg/kg)	Harlan-Teklad	Cat# TD.01306
Uracil-DNA glycosylase	New England Biolabs	Cat# M0280L
Eosin Y disodium salt	Sigma-Aldrich	Cat# E6003-25G
Nigrosin	Sigma-Aldrich	Cat# 198285-25G
Human chorionic gonadotropin	Sigma-Aldrich	Cat# CG10
CARD MEDIUM set (includes FERTIUP)	CosmoBioUSA	Cat# KYD-005-EX
CARD HyperOva	CosmoBioUSA	Cat# KYD-010-EX-X5
Mineral oil	Sigma-Aldrich	Cat# M5310
BSA (Fraction V, fatty acid-free) embryo tested lot	Sigma-Aldrich	Cat# A3311
Sodium lactate (60% w/w)	Sigma-Aldrich	Cat# L7900
Glucose (D+)	Sigma-Aldrich	Cat# P4562
Penicillin G	Sigma-Aldrich	Cat# P7794
Streptomycin	Sigma-Aldrich	Cat# S1277
MluI-HF	New England Biolabs	Cat# R3198S
Phusion high fidelity DNA polymerase	New England Biolabs	Cat# M0530S
APOBEC3A-MycHis	Stenglein et al.^62^	N/A

**Critical commercial assays**		

RNeasy Mini Kit	Qiagen	Cat# 74104
Qiashredder	Qiagen	Cat# 79654
DNeasy Blood & Tissue Kit	Qiagen	Cat# 69506
Bradford Protein Assay	Bio-Rad	Cat# 5000001

**Deposited data**		

WGS data	This paper	Sequence Read Archive # PRJNA927047
RNA-seq data	This paper	Sequence Read Archive # PRJNA927047

**Experimental models: Cell lines**		

293T	American Type Culture Collection	Cat# CRL-3216 RRID: CVCL_0063
KH2 ES cells	Gifted by Dr. Sagrario Ortega	Beard et al.^63^

**Experimental models: Organisms/strains**		

Mouse: B6.C-Tg(CMV-cre)1Cgn/J	The Jackson Laboratory	Strain# 006054 RRID: IMSR_JAX:006054
Mouse: B6.Tg(MMTV-cre)4Mam/J	The Jackson Laboratory	Strain# 003553 RRID: IMSR_JAX:003553
Mouse: B6.*Rosa26::LSL-A3Bi*	Boumelha et al.^15^	N/A
Mouse: B6.*Rosa26::CAG-LSL-A3Bi*	This paper	Donated to Jackson Laboratory: Strain# 038176
Mouse: B6.*Rosa26::CAG-LSL-A3Bi-E255A*	This paper	Donated to Jackson Laboratory: Strain# 038177
Mouse: B6.Cg-*Gt(ROSA)26Sor*^*tm1(rtTA∗M2)Jae*^/J	The Jackson Laboratory	Strain# 006965 RRIS: IMSR_JAX:006965
Mouse: B6.*Col1a1::TetO-A3B-tGFP*	This paper and de la Vega et al.^20^	N/A

**Oligonucleotides**		

Table S3	Integrated DNA Technologies	N/A

Recombinant DNA		

*Col1a1::TetO-A3B-tGFP*	This paper and de la Vega et al.^20^	N/A
*R26::CAG-LSL-A3Bi*	This study	N/A
*R26::LSL-A3Bi*	Boumelha et al.^15^	N/A
*R26::CAG-LSL-A3Bi-E255A*	This paper	N/A
pcDNA3.1-A3Ai-Myc-His	Stenglein et al.^62^	N/A
pAi38	Addgene	RRID: Addgene_34883
pEGFP-N3-A3Bi-E255A	This paper	N/A

**Software and algorithms**		

SAS v9.4	SAS Institute	https://www.sas.com/en_us/software/stat.html
GraphPad Prism v9.4	GraphPad software	http://www.graphpad.com
QuPath v0.4.2	Bankhead et al.^64^	https://qupath.github.io
NIS Elements v4.11.0	NIS-Elements Viewer software	https://www.microscope.healthcare.nikon.com/products/software/nis-elements/viewer
STAR/2.7.10a	Dobin et al.^65^	https://github.com/alexdobin/STAR/releases
Picard tools v2.18.16	Broad Institute	https://broadinstitute.github.io/picard/
TRUST4 v1.0.8	Song et al.^66^	https://github.com/liulab-dfci/TRUST4
HISAT2	Kim et al.^67^	https://github.com/DaehwanKimLab/hisat2
Cufflinks	Trapnell et al.^68^	https://github.com/cole-trapnell-lab/cufflinks
deconstructSigs v1.8.0	Rosenthal et al.^69^	https://github.com/raerose01/deconstructSigs
Trimmomatic v0.33	Bolger et al.^70^	https://github.com/timflutre/trimmomatic
SpeedSeq v0.1.2	Chiang et al.^71^	https://github.com/hall-lab/speedseq
GATK3 v3.6.0	Broad Institute	https://gatk.broadinstitute.org/hc/en-us
MutationalPatterns v3.17	Blokzijl et al.^72^	https://github.com/ToolsVanBox/MutationalPatterns
SigProfilerTopography v.1.0.70	SigProfiler Bioinformatic Tools	https://github.com/AlexandrovLab/SigProfilerTopography
lsa v0.73.3	CRAN R Project for Statistical Computing	https://CRAN.R-project.org/package=lsa
Pyclone-VI v0.1.1	Gillis et al.^35^	https://github.com/Roth-Lab/pyclone-vi
CNVKit v0.9.10	Talevich et al.^73^	https://github.com/etal/cnvkit
Manta v1.6.0	Chen et al.^74^	https://github.com/Illumina/manta

**Other**		

Typhoon FLA-7000 Image Reader	GE Life Sciences	N/A
Odyssey Fc	Li-COR	N/A
Odyssey Classic	Li-COR	N/A
NovaSeq 6000	Illumina	N/A
Aperio AT2	Aperio	N/A
Reichert-Jung BioCut 2030 Rotary Microtome	Leica	N/A
Huron TissueScope LE	Huron Digital Pathology	N/A
C2 DS-Ri1	Nikon	N/A
MC170 HD	Leica	N/A
DM IRE2	Leica	N/A
Branson 450 Analog Sonifier	Branson	N/A

### Resource availability

#### Lead contact

Requests for additional data needed to recapitulate results reported in the paper should be directed to the lead contact: Reuben S. Harris (rsh@uthscsa.edu).

#### Materials availability

All materials generated in this study are available through the lead contact. *Rosa26::CAG-LSL-A3Bi* and *Rosa26::CAG-A3Bi-E255A* mice generated here are also available through Jackson Laboratory as strains #038176 and #038177, respectively.

### Experimental models and subject details

#### Animal experiments

C57BL/6 animals were used to generate genetically engineered mice at the Gene Targeting & Transgenic Facility at the HHMI Janelia Campus (B6.*Rosa26::CAG-LSL-A3Bi* and B6.*Rosa26::CAG-LSL-A3Bi-E255A*) or R. Sotillo’s laboratory (B6.*Col1a1::TetO-A3B-tGFP*). Other experimental mice were purchased from The Jackson Laboratories. All mice were housed in specific pathogen-free conditions at 22*°*C under a standard 12 h light/dark cycle, and handled in agreement with local Animal Care and Ethics committees. All mice were fed standard laboratory chow, with the exception of B6.*Col1a1::TetO-A3B-tGFP* mice, which were fed food pellets containing doxycycline (625 mg/kg; Harlan-Teklad) to induce A3B-tGFP expression. Mice were housed at the University of Minnesota Twin Cities and University of Texas Health San Antonio animal facilities in specific pathogen-free conditions in accordance with the Institutional Animal Care and Use Committee guidelines (protocol 2201-39748A and 20220024AR, respectively). Mouse experiments performed in DKFZ animal facilities had ethical approval from Baden-Wurttemberg, Germany (license number G-29-19). Murine experiments at the University of Oslo, Institute of Basal Medical Sciences animal facilities were done in minimal disease units and had ethical approval from the Norwegian Food Safety Authority (FOTS ID7569). Both male and female mice were used for all experiments, except for B6.Tg(MMTV-cre)4Mam/J *Rosa26::CAG-LSL-A3Bi* mice, which were exclusively female. Mice of both genders developed tumors, and both male and female mice were analyzed using downstream processes including whole-genome sequencing and histopathological analysis. We concluded that gender does not affect tumor development in this study, although males are infertile. WT and B6.*Rosa26::LSL-A3Bi* mice were reported previously,^15^ although a more in-depth tumor analysis is provided here. Additionally, mouse tumor-free survival here only reports animals that were euthanized due to poor body condition.

#### Human tumor specimens

Additional slices of previously reported,^61^ archived formalin-fixed paraffin-embedded (FFPE) human tissue specimens (N = 7) were obtained from the Oral Pathology Laboratory, School of Dentistry, University of Minnesota following institutional review board approval. Informed consent was obtained from all patients prior to tissue procurement for diagnostic purposes. Specimens comprised incisionally or excisionally biopsied oral or oropharyngeal epithelial lesions diagnosed as follows: HPV-positive oral epithelial dysplasia (N = 2), HPV-positive oropharyngeal squamous cell carcinoma (SCC; N = 1), and HPV-negative oral SCC (N = 4). Independent A3B IHC analyses of these samples were reported previously.^61^ Information regarding the age, gender and smoking history of the patients, anatomic site, histopathologic diagnosis, and p16 status of the lesions can be found in Table S2.

#### Cell culture

293T cells were purchased from ATCC and KH2 ES cells were a gift from Dr. Sagrario Ortega.^63^ Cell lines were cultured at 37°C and 5% CO_2_. 293T cells were cultured in DMEM (Gibco) supplemented with 10% FBS (Gibco) and penicillin-streptomycin (Gibco). Transfection was performed using TransIT-LT1 (Mirus Bio Corporation). KH2 ES cells were cultured in ES-KO media supplemented with FBS, MEM with Glutamax (Gibco), NEAA non-essential amino acids (Gibco), penicillin-streptomycin, 2-mercaptoetanol (Gibco), and Leukemia Inhibitory Factor. Electroporation was performed using the Neon Transfection system (Thermo Fisher Scientific) with 5 μg of TRE-A3B-tGFP vector and 2.5 μg of the pCAG-flpE-puro vector following the manufacturer’s instructions. After hygromycin B selection, positive clones were then picked and expanded. These cells were confirmed to contain the A3B-tGFP transgene by genotyping and were then used for implantation.

### Method details

#### A3B knock-in

A description of the *Rosa26::LSL-A3Bi* knock-in has been published.^15^ A *Rosa26::CAG-LSL-A3Bi* targeting construct was generated using pAi38 (Addgene) as a backbone, which contains the strong chimeric CAG (cytomegalovirus early enhancer/chicken *β-actin*/rabbit *β-globin* 3′ splice acceptor) promoter and *Rosa26* targeting arms. The plasmid was cut and a hybridized *loxP* oligo pair was ligated (RSH13242 and RSH13243) to this backbone to make an intermediate plasmid. A fragment containing the NEO-stop-loxP-A3Bi elements from the *Rosa26::LSL-A3Bi* plasmid was cut and ligated into the backbone of the intermediate plasmid to create the final *Rosa26::CAG-LSL-A3Bi* construct. To generate the *Rosa26::CAG-LSL-A3Bi-E255A* targeting construct, *A3Bi-E255A* was amplified from a separate *in house* plasmid using Phusion polymerase (New England Biolabs) and primers with the MluI-HF restriction site RSH13293 and RSH13294. Both the *Rosa26::CAG-LSL-A3Bi* plasmid and the amplicon were cut with MluI-HF (New England Biolabs), then ligated together to create the targeting construct. Final constructs were used for a targeted knock-in into the *Rosa26* locus of C57BL/6 embryonic stem cells at the Gene Targeting & Transgenic Facility at the Howard Hughes Medical Institute Janelia Research Institute. B6.*Col1a1::TetO-A3B-tGFP* mice were generated using KH2 ES cells (gifted by Dr. Sagrario Ortega), which carry the M2-rtTA gene inserted within the *Rosa26* allele.^63^ A construct containing the human *A3B-tGFP* cDNA under the control of the tetracycline response element (TRE) was inserted downstream of the *Col1a1* locus.^20^

#### Mouse procedures

Female B6.C-Tg(CMV-cre)1Cgn/J mice (Jax strain 006054)^75^ were crossed with male B6.*Rosa26::LSL-A3Bi*,^15^ B6.*Rosa26::CAG-LSL-A3Bi*, and B6.*Rosa26::CAG-LSL-A3Bi-E255A* animals. The resulting pups with *CMV-Cre* and an excised *loxP*-STOP-*loxP* cassette (*i.e*., single *loxP* site) were crossed with WT C57BL/6 animals to obtain experimental animals with or without full-body A3B expression. For mammary duct expression of A3B, B6.*Rosa26::CAG-LSL-A3Bi* mice were crossed with B6.Tg(MMTV-cre)4Mam/J mice (Jax strain 003551). The resulting pups were genotyped, enrolled, and monitored weekly and aged out until tumors could be observed either visually or by palpation. Genomic DNA was isolated using the Gentra Puregene protocol (Qiagen) on mouse tail biopsies from animals at 21 days of age, with 50 ng of DNA used as a PCR template. A genotyping schematic for B6.*Rosa26::LSL-A3Bi* and B6.*Rosa26::CAG-LSL-A3Bi* mice is provided in Figure S1. The B6.*Rosa26::CAG-LSL-A3Bi* genotyping schematic can be used for B6.*Rosa26::CAG-LSL-A3Bi-E255A* mice. Due to upstream MluI restriction site removal when generating *Rosa26::CAG-LSL-A3Bi-E255A* constructs, mice can be differentiated by cutting PCR amplicons with MluI (New England Biolabs) or by Sanger sequencing. All oligonucleotides used for genotyping in this study can be found in Table S3 along with their target alleles. For our original experiment (Figure 3), WT control mice were littermates of B6.*Rosa26::LSL-A3Bi* animals. Due to COVID-19-based experimental restrictions, WT littermates of B6.*Rosa26::CAG-LSL-A3Bi* were not used, and B6.*Rosa26::CAG-L-A3Bi* mice were euthanized at a set timepoint of 547 days. For the second experiment (Figure 6), WT control mice were littermates of both B6.*Rosa26::CAG-L-A3Bi* and B6.*Rosa26::CAG-L-A3Bi-E255A* mice. Mice were monitored three times a week for signs of excessive pain or discomfort, or until their tumors reached >1 cm^3^. All mice were euthanized via CO_2_ asphyxiation, then control tissues and tumors were immediately collected to be fixed in buffered 10% formalin or flash-frozen in liquid nitrogen. Tumors were initially scored based on visual diagnosis, and then subsequently confirmed with histopathological analysis.

#### Immunoblots

Tissue lysates were homogenized, lysed, and quantified as above, and then treated with an equal amount of SDS-PAGE loading buffer (62.5 mM Tris-Cl, pH 6.8, 20% glycerol, 7.5% SDS, 5% 2-mercaptoethanol, and 250 mM DTT) and denatured by heating at 95°C. Proteins were then separated using an SDS-PAGE gel and transferred to a polyvinylidene Immobilon-FL membrane. Membranes were washed in PBS, then soaked in 5% milk + PBST to block nonspecific binding. The membranes were incubated in a primary rabbit α-human A3A/B/G antibody 1:1,000 (Brown et al.) and mouse α-tubulin 1:10,000 (Sigma Aldrich), or rabbit α-actin 1:5,000 (Sigma Aldrich) at 4°C overnight. Membranes were then washed in PBST six times for 5 min each, then incubated for 1 h with secondary α-rabbit HRP (Cell Signaling Technology) and goat α-mouse 800 (LI-COR) at 1:10,000 dilutions supplemented with 0.02% SDS. These membranes were then washed 5 times in PBST and one time in PBS for 5 min each, then imaged using an Odyssey Classic scanner and Odyssey Fc imager (LI-COR).

#### DNA deaminase activity assays

Tissues from animals were homogenized and lysed in HED buffer (25 mM HEPES, 5 mM EDTA, 10% glycerol, 1 mM DTT, and 1x cOmplete protease inhibitor [Roche]). Lysates were sonicated for 20 min in a water bath sonicator and cleared by centrifugation. Protein concentration was quantified using a Bradford Assay (Bio-Rad) and was normalized to the same amount for the assay. Samples were incubated for 1 h at 37°C with the HED buffer solution supplemented with 100 μg/mL RNase A (Qiagen), 0.1 U of uracil DNA glycosylase (New England Biolabs), 100 μM RSH 5194 and 1x UDG buffer (New England Biolabs).^60^ Sodium hydroxide was added to make a 100 mM concentration solution, and incubated at 98°C for 10 min, followed by the addition of 1x formamide buffer (80% formamide, 90 mM Tris, 90 mM Boric acid, 2 mM EDTA) and a subsequent 98°C incubation for 10 min. Samples were run on a 15% TBE-Urea gel and imaged using a Typhoon 7000 FLA biomolecular imager (GE Healthcare Life Sciences).

#### Protein purification

Purification of A3A-MycHis as a positive control for deaminase activity assays was performed similar to as previously described.^62^ First, 293T cells were transfected with pcDNA3.1-A3A-Myc-His. 48 h later, 1 × 10^8^ cells were harvested, washed with PBS, and resuspended in 10 mL of cell lysis buffer (25 mM HEPES, pH7.4, 300 mM NaCl, 20 mM imidazole, 0.1% triton X-100, 10 mM MgCl_2_, 0.5% TCEP, and 10% glycerol). The cell suspension was sonicated using a Branson Sonifier 450 for 2 min at 40% duty cycle power 5. RNase A (Qiagen) and Benzonase were added to the suspension to 100 μg/mL and 5 μL/25 mL, respectively. These were then incubated at 37°C for an hour with nucleases and subsequently clarified by spinning at 16,000 g for 30 min at room temperature. NaCl was then added to these lysates to a final concentration of 1M. 50 μL of Nickel-NTA (Qiagen) superflow beads were added and mixed by rotating for 2 h at room temperature. This was then loaded onto a Poly-Prep chromatography column (Bio-Rad) and washed using wash buffer (25 mM HEPES, pH 7.4, 300 mM NaCl, 0.1% triton X-100, 40 mM imidazole, and 20% glycerol). The final protein was eluted using elution buffer (25 mM HEPES, pH 7.4, 300 mM NaCl, 0.1% triton X-100, 300 mM imidazole, 20% glycerol, and 1 mM TCEP) and concentration was determined using Bradford assays (Bio-Rad).

#### Immunohistochemistry (IHC)

IHC was performed as described.^17^^,^^76^ Formalin-fixed paraffin-embedded tissues were sectioned into 4 μm slices using a Reichert-Jung BioCut 2030 Rotary Microtome and mounted on positively charged adhesive slides. They were then baked at 65°C for 20 min to deparaffinize them, and rehydrated with three consecutive washes in CitriSolv (Decon Labs) for 5 min each followed by graded alcohols as above, followed by a final 5 min wash in running water. Epitope retrieval was performed with the Reveal Decloaker (BioCare Medical) by steaming for 35 min with a subsequent 30 min off the steamer. Then, slides were washed for 5 min with running water followed by Tris-buffered saline with 0.1% Tween 20 (TBST) for 5 min. To suppress endogenous peroxidase activity, the slides were soaked in 3% H_2_O_2_ diluted in TBST for 10 min, followed by a 5 min rinse in running water. A 15 min soak in Background Sniper (BioCare Medical) was used to block nonspecific binding, with an immediate successive overnight incubation with primary antibody diluted in 10% Sniper in TBST at 4°C. Primary antibodies used for detection were CD3 (Abcam) at a 1:300 dilution, α-A3B/A/G 5210-87-13 (Brown et al.) at a 1:350 dilution, B220 (BD Pharmogen) at a 1:100 dilution, and γ-H2AX (Cell Signaling) at a 1:200 dilution. Following overnight incubation with primary antibody, samples were washed with TBST for 5 min, then incubated for 30 min with Novolink Polymer (Leica Biosystems). This was developed by application of the Novolink DAB substrate kit (Leica Biosystems) for 5 min, then it was rinsed in water for 5 min, and counterstained for 10 min using Mayer’s hematoxylin solution (Electron Microscopy Sciences). These were rinsed in tap water for 10 min then dehydrated in graded alcohols and CitriSolv, and cover-slipped with Permount mounting media (Thermo Fisher Scientific).

#### Hematoxylin and eosin (H&E) staining

All tissues were fixed overnight in 10% buffered formalin, and then embedded in paraffin. Fixed tissues were then sectioned into 4 μm slices using a Reichert-Jung BioCut 2030 Rotary Microtome and mounted onto positively charged adhesive glass slides. After air-drying, they were baked at 60°C–62*°*C for 20 min, washed with xylene for 5 min 3 times, soaked in graded alcohols (100% x 2, 95% x 1 and 80% x 1) for 3 min each, and finally rinsed in tap water for 5 min. They were then stained with hematoxylin for 5 min and rinsed in tap water for 30 s, followed by brief submersion in an acid solution and 30–90 s in ammonia water. They were then washed with water for 10 min, 80% ethanol for 1 min, counterstained with eosin for 1 min, dehydrated in graded alcohols followed by xylene as above, and coverslipped with Cytoseal (Thermo Fisher Scientific).

#### Eosin-nigrosin staining

For male sterility experiments, 2-month-old littermate male mice were euthanized and relavent reproductive tissues were collected. Sperm were obtained by incubating a lacerated cauda epididymis in a pre-warmed HEPES-0.1% BSA buffer consisting of 130mM NaCl, 4mM KCl, 14mM fructose, 10mM HEPES, 1.35mM CaCl_2_, 1 mM MgCl_2_ in a droplet covered by embryo tested neat mineral oil. After incubating the cauda epididymis at 36°C for 30 min to allow the sperm cells to swim out, they were stained for a morphological and quantitative viability analysis. To stain the above sperm cells for a morphological and quantitative viability analysis, eosin-nigrosin staining was performed by using two parts 1% eosin Y (Sigma-Aldrich) and two parts 10% nigrosin (Sigma-Aldrich) well-mixed with one part mouse sperm cells. The resulting mix was then smeared on slides, and a coverslip applied with Cytoseal mounting media. Photographs of these slides were taken using a Nikon C2 DS-Ri1 color camera and analyzed using NIS Elements Viewer.

#### *In vitro* fertilization

For all *in vitro* fertilization experiments, a modified version of the Nakagata method was followed.^77^ Briefly, superovulation was induced in female mice 23–26 days old by injecting 0.1 mL of HyperOva (CosmobioUSA) per female at 6:00 p.m., followed by an injection of 5 IU human chorionic gonadotropin (Sigma-Aldrich) 47 h later. 15 h following human chorionic gonadotropin injection, 100μL of FERTIUP (CosmoBioUSA) was covered with embryo tested neat mineral oil in sterile Petri plates and incubated for at 36°C for 30 min. Fresh sperm were obtained from 2-month-old male mice by transferring sperm directly from a lacerated cauda epididymis in mineral oil to FERTIUP. Sperm were allowed to disperse in the FERTIUP medium for 60 min in a 36°C 5% CO_2_ incubator. 200 μL of CARD MEDIUM (CosmoBioUSA) was put in a Petri dish, covered with neat mineral oil, and incubated at 36°C 5% CO_2_ for 10 min. Satisfactory sperm motility and number were confirmed before fertilization. Oviducts from female mice were dissected, and cumulus-oocyte complexes (COC) were collected from swollen ampulla and put into CARD MEDIUM. Fertilization was induced by aspirating 10 mL of the incubated FERTIUP sperm suspension and adding it to the COC CARD MEDIUM. 3 h post-insemination of sperm-COCs, presumptive zygotes were washed 3x in filter-sterilized modified high calcium HTF (100mM NaCL, 5mM KCl, 200μM MgSO_4_ · 7H_2_O, 400 μM KH_2_PO_4_, 5 mM CaCl_2_ · 2H_2_O, 25 mM NaHCO_3_, 2.8 mM Glucose [D+], 200 μM Penicillin G, 70 μM Streptomycin, 60 mM BSA, and 340 mL 60% w/w Sodium Lactate) pre-warmed to 36°C, and then incubated in high calcium HTF. Developing embryos were quantified and imaged using a Leica DM IRE2 microscope with the Leica MC170 HD camera.

#### IHC quantification

Whole-slide images of the murine tissues or human oral/oropharyngeal lesions immunostained with the α-A3A/B/G rabbit mAb (5210-87-13) were generated at 20-40× magnification using an Aperio AT2 or a Huron TissueScope LE microscope slide scanner and analyzed using QuPath software.^64^ A3B histoscore (H-score) calculations were performed using the QuPath nuclear staining algorithm. This algorithm identifies cell nuclei in designated lesional areas and quantifies staining intensity as follows: negative, weak, moderate, or strong. A3B H-score was calculated for each lesion via the linear formula: H-score = 1x(%weak-positive cells) + 2x(%moderate-positive cells) + 3x(%strong-positive cells) as described.^16^^,^^61^^,^^76^ Areas for analysis containing lesional tissue were determined using H&E-stained slides, and H-scores were calculated using these areas exclusively.

#### DNA and RNA extraction and sequencing

Genomic DNA was prepared for sequencing from frozen tissues using the DNeasy Blood and Tissue Kit (Qiagen), and RNA was extracted from frozen tissues using the RNeasy Mini kit (Qiagen). In both cases, tissue were homogenized using Qiashredder columns (Qiagen). For DNA sequencing, libraries were sequenced 2 × 150 paired end using a NovaSeq 6000 instrument (Illumina) to get 30x coverage on the genome. Similarly for RNA sequencing, libraries were sequenced 2 × 150 paired end on a NovaSeq 6000 to get 20 million reads per sample. Raw sequence reads were aligned to the mm10 reference mouse genome. All WGS and RNAseq reactions were done at the University of Minnesota Genomics Center.

#### RNA sequencing analysis

RNA-seq reads were aligned to mouse genome mm10 using STAR/2.7.1a with basic two pass mode for realigning splice junctions enabled.^65^ Picard tools (version 2.18.16) were then used to mark duplicate reads, split CIGAR reads with Ns at the splice junctions. Immune repertoire of lymphomas (V(D)J enrichment) were reconstructed using TRUST4 following its suggested pipeline.^66^ RNA-seq expression levels were calculated using HISAT2^67^ and Cufflinks.^68^ Mutect2 from GATK (3.6) was used to call RNA edits relative to the matched normal RNA-seq data. Editing events that passed the Mutact2 internal filter with at least 3 reads supporting the edit and a minimum of 10 total reads at the editing site and a variant allele frequency greater than 0.05 were compared to the matched tumor DNA somatic mutations. Editing events that were not seen in the matched tumor DNA were used for downstream analysis. MutationalPatterns R package and SigProfiler tools were used to analyze mutation signatures.

#### Whole genome sequencing analysis

Whole genome sequencing reads were trimmed to remove low quality reads and adapter sequences using Trimmomatic v0.33.^70^ Trimmed reads were aligned to the mouse genome (mm10) using SpeedSeq.^71^ PCR duplicates were removed using Picard (version 2.18.16). Reads were locally realigned around indels using GATK3 (version 3.6.0) tools. Single base substitutions and small indels were called relative to the matched normal tissues using Mutect2. SBSs that passed the internal GATK3 filter with minimum 3 reads supporting each variant, minimum 10 total reads at each variant site and a variant allele frequency over 0.05 were used for downstream analysis. Somatic structural variations were detected using Manta following the somatic structural variation described by Manta using sorted and indexed tumor and matched normal bam files.^74^ Bioconductor package MutationalPatterns was used to plot the mutation and indel landscapes and extract *de novo* signatures using non-negative matrix factorization.^72^ R package lsa (version 0.73.3) was used to calculate cosine similarity of *de novo* signatures to Cosmic signatures. SigProfiler tools were used to further analyze mutations including replication timing. PyClone-VI (version 0.1.1) was used to determine subclonal heterogeneity, with input files from each tumor containing single nucleotide variations called by Mutect2 and copy number variations determined by CNVkit (version 0.9.10).^35^^,^^73^ Subclones were defined as clusters identified by PyClone-VI with low cellular prevalence (<1%) clusters excluded.

#### Somatic mutation analysis

APOBEC enrichment scores were calculated in the R statistical language (version 4.1.2) using variant calls from the sequencing data. First, the data were organized by 1) filtering for single-base substitutions, 2) filtering for C:G base pairs in the reference sequence, 3) removing mutations derived from the mitochondrial genome, and 4) removing C-to-A substitutions, which are confounded by other mutagenic processes. Next, a 41-base sequence context, consisting of 20 bases up- and downstream of the mutated position, was extracted from the mm10 reference genome (*BSgenome.Mmusculus.UCSC.mm10* package; version 1.4.3). Finally, APOBEC enrichment scores were computed using the following formula:

$${APOBEC\;Enrichment}_{TCW}=\frac{{Mut}_{TCW}\;/\;{Con}_{TCW}}{{Mut}_{C}\;/\;{Con}_{C}}$$

TCW represents the sequence motifs (TCA and TCT) preferred by APOBEC enzymes for cytidine deamination. Mut_TCW_ represents the total number of mutated cytosines in the TCW motif in the 41-base window. Mut_C_ represents the total number of mutated cytosines in the 41-base window. Con_TCW_ and Con_C_ are the total numbers of TCW motifs or cytosines in the 41-base window, respectively. Calculations for the terms above were made for each substitution, and the values were aggregated prior to computing the APOBEC enrichment score for each sample. Statistical significance was calculated using a one-sided Fisher exact test comparing the Mut_TCA_/(Mut_C_ – Mut_TCA_) and the Con_TCW_/(Con_C_ – Con_TCW_) ratios. P values were adjusted using Benjamini-Hochberg correction. The percent contribution of each single-base substitution (SBS) signature (SBS1 to SBS30) was calculated using variant calls from the sequencing data. The *whichSignatures* function in the *deconstructSigs* package (version 1.8.0) in R was applied.^69^

### Quantification and statistical analysis

Time to tumor formation was summarized using Kaplan-Meier curves and compared across groups using log rank tests. Correlative statistical analyses were performed using Pearson correlation coefficient or Spearman’s rank correlation coefficient and were considered significant if the corresponding p value was <0.05. For statistical analyses to test the outcome between two groups, the median total values were compared by group using Mann-Whitney U test as they have non-normal distributions. To compare two independent categorical groups, the Fisher’s Exact test was used. To compare more than two independent categorical groups, the chi-square test was used. Details for each analysis including test, p value, and number analyzed can be found in the figure legends or figures themselves. Data were analyzed using SAS 9.4 (Cary, NC) and GraphPad Prism 9.4. P values <0.05 (or false discovery rate q values <0.1 for high APOBEC enrichment scores) were considered statistically significant.

## Data Availability

• Whole genome sequencing and RNA-seq data have been uploaded to the Sequence Read Archive at the National Library of Medicine and are available publicly as project #PRJNA927047. Original immunoblot images reported in this paper will be shared by the lead contact upon request. • No original code is reported here. • Any additional information required to reanalyze the data reported in this paper is available from the lead contact upon request.

## Supplemental information

Document S1. Figures S1–S7

Table S1. CAG-A3B and WT animal information, related to Figure 3

Table S2. Information for HNSCC tumors used for IHC studies, related to Figure 3

Table S3. Oligonucleotide sequences, related to STAR Methods

Document S2. Article plus supplemental information

## References

[1] Hanahan D. (2022). Hallmarks of cancer: new dimensions. Cancer Discov..

[2] Persi E., Wolf Y.I., Horn D., Ruppin E., Demichelis F., Gatenby R.A., Gillies R.J., Koonin E.V. (2021). Mutation–selection balance and compensatory mechanisms in tumour evolution. Nat. Rev. Genet..

[3] Reiter J.G., Baretti M., Gerold J.M., Makohon-Moore A.P., Daud A., Iacobuzio-Donahue C.A., Azad N.S., Kinzler K.W., Nowak M.A., Vogelstein B. (2019). An analysis of genetic heterogeneity in untreated cancers. Nat. Rev. Cancer.

[4] Koh G., Degasperi A., Zou X., Momen S., Nik-Zainal S. (2021). Mutational signatures: emerging concepts, caveats and clinical applications. Nat. Rev. Cancer.

[5] Alexandrov L.B., Kim J., Haradhvala N.J., Huang M.N., Tian Ng A.W., Wu Y., Boot A., Covington K.R., Gordenin D.A., Bergstrom E.N. (2020). The repertoire of mutational signatures in human cancer. Nature.

[6] Saini N., Gordenin D.A. (2020). Hypermutation in single-stranded DNA. DNA Repair.

[7] Harris R.S., Dudley J.P. (2015). APOBECs and virus restriction. Virology.

[8] Pecori R., Di Giorgio S., Paulo Lorenzo J., Nina Papavasiliou F. (2022). Functions and consequences of AID/APOBEC-mediated DNA and RNA deamination. Nat. Rev. Genet..

[9] Swanton C., McGranahan N., Starrett G.J., Harris R.S. (2015). APOBEC enzymes: mutagenic fuel for cancer evolution and heterogeneity. Cancer Discov..

[10] Carpenter M.A., Temiz N.A., Ibrahim M.A., Jarvis M.C., Brown M.R., Argyris P.P., Brown W.L., Yee D., Harris R.S., Harris R.S. (2023). Mutational impact of APOBEC3A and APOBEC3B in a human cell line and comparisons to breast cancer. bioRxiv.

[11] Petljak M., Dananberg A., Chu K., Bergstrom E.N., Striepen J., von Morgen P., Chen Y., Shah H., Sale J.E., Alexandrov L.B. (2022). Mechanisms of APOBEC3 mutagenesis in human cancer cells. Nature.

[12] Salas-Briceno K., Zhao W., Ross S.R. (2020). Mouse APOBEC3 restriction of retroviruses. Viruses.

[13] Law E.K., Levin-Klein R., Jarvis M.C., Kim H., Argyris P.P., Carpenter M.A., Starrett G.J., Temiz N.A., Larson L.K., Durfee C. (2020). APOBEC3A catalyzes mutation and drives carcinogenesis *in vivo*. J. Exp. Med..

[14] Naumann J.A., Argyris P.P., Carpenter M.A., Gupta H.B., Chen Y., Temiz N.A., Zhou Y., Durfee C., Proehl J., Koniar B.L. (2023). DNA deamination is required for human APOBEC3A-driven hepatocellular carcinoma *in vivo*. Int. J. Mol. Sci..

[15] Boumelha J., de Carné Trécesson S., Law E.K., Romero-Clavijo P., Coelho M.A., Ng K.W., Mugarza E., Moore C., Rana S., Caswell D.R. (2022). An immunogenic model of KRAS-mutant lung cancer enables evaluation of targeted therapy and immunotherapy combinations. Cancer Res..

[16] Argyris P.P., Naumann J., Jarvis M.C., Wilkinson P.E., Ho D.P., Islam M.N., Bhattacharyya I., Gopalakrishnan R., Li F., Koutlas I.G. (2023). Primary mucosal melanomas of the head and neck are characterised by overexpression of the DNA mutating enzyme APOBEC3B. Histopathology.

[17] Brown W.L., Law E.K., Argyris P.P., Carpenter M.A., Levin-Klein R., Ranum A.N., Molan A.M., Forster C.L., Anderson B.D., Lackey L., Harris R.S. (2019). A rabbit monoclonal antibody against the antiviral and cancer genomic DNA mutating enzyme APOBEC3B. Antibodies.

[18] Land A.M., Law E.K., Carpenter M.A., Lackey L., Brown W.L., Harris R.S. (2013). Endogenous APOBEC3A DNA cytosine deaminase is cytoplasmic and nongenotoxic. J. Biol. Chem..

[19] Salamango D.J., McCann J.L., Demir Ö., Brown W.L., Amaro R.E., Harris R.S. (2018). APOBEC3B nuclear localization requires two distinct N-terminal domain surfaces. J. Mol. Biol..

[20] Alonso de la Vega A., Temiz N.A., Tasakis R., Somogyi K., Reuveni E., Ben-David U., Stenzinger A., Poth T., Papavasiliou N., Harris R.S., Sotillo R. (2022). Acute expression of human APOBEC3B in mice causes lethality associated with RNA editing. bioRxiv.

[21] Ewald D., Li M., Efrat S., Auer G., Wall R.J., Furth P.A., Hennighausen L. (1996). Time-sensitive reversal of hyperplasia in transgenic mice expressing SV40 T antigen. Science.

[22] Hennighausen L., Wall R.J., Tillmann U., Li M., Furth P.A. (1995). Conditional gene expression in secretory tissues and skin of transgenic mice using the MMTV-LTR and the tetracycline responsive system. J. Cell. Biochem..

[23] Wagner K.-U., Wall R.J., St-Onge L., Gruss P., Wynshaw-Boris A., Garrett L., Li M., Furth P.A., Hennighausen L. (1997). Cre-mediated gene deletion in the mammary gland. Nucleic Acids Res..

[24] Nik-Zainal S., Van Loo P., Wedge D.C., Alexandrov L.B., Greenman C.D., Lau K.W., Raine K., Jones D., Marshall J., Ramakrishna M. (2012). The life history of 21 breast cancers. Cell.

[25] Nik-Zainal S., Alexandrov L.B., Wedge D.C., Van Loo P., Greenman C.D., Raine K., Jones D., Hinton J., Marshall J., Stebbings L.A. (2012). Mutational processes molding the genomes of 21 breast cancers. Cell.

[26] Alexandrov L.B., Nik-Zainal S., Wedge D.C., Aparicio S.A.J.R., Behjati S., Biankin A.V., Bignell G.R., Bolli N., Borg A., Børresen-Dale A.L. (2013). Signatures of mutational processes in human cancer. Nature.

[27] Mas-Ponte D., Supek F. (2020). DNA mismatch repair promotes APOBEC3-mediated diffuse hypermutation in human cancers. Nat. Genet..

[28] Roberts S.A., Sterling J., Thompson C., Harris S., Mav D., Shah R., Klimczak L.J., Kryukov G.V., Malc E., Mieczkowski P.A. (2012). Clustered mutations in yeast and in human cancers can arise from damaged long single-strand DNA regions. Mol. Cell.

[29] Kazanov M.D., Roberts S.A., Polak P., Stamatoyannopoulos J., Klimczak L.J., Gordenin D.A., Sunyaev S.R. (2015). APOBEC-induced cancer mutations are uniquely enriched in early-replicating, gene-dense, and active chromatin regions. Cell Rep..

[30] Aaltonen L.A., Abascal F., Abeshouse A., Aburatani H., Adams D.J., Agrawal N., Ahn K.S., Ahn S.-M., Aikata H., ICGC/TCGA Pan-Cancer Analysis of Whole Genomes Consortium (2020). Pan-cancer analysis of whole genomes. Nature.

[31] Christensen S., Van Der Roest B., Besselink N., Janssen R., Boymans S., Martens J.W.M., Yaspo M.-L., Priestley P., Kuijk E., Cuppen E., Van Hoeck A. (2019). 5-fluorouracil treatment induces characteristic T>G mutations in human cancer. Nat. Commun..

[32] Nik-Zainal S., Kucab J.E., Morganella S., Glodzik D., Alexandrov L.B., Arlt V.M., Weninger A., Hollstein M., Stratton M.R., Phillips D.H. (2015). The genome as a record of environmental exposure. Mutagenesis.

[33] Behjati S., Huch M., Van Boxtel R., Karthaus W., Wedge D.C., Tamuri A.U., Martincorena I., Petljak M., Alexandrov L.B., Gundem G. (2014). Genome sequencing of normal cells reveals developmental lineages and mutational processes. Nature.

[34] Riva L., Pandiri A.R., Li Y.R., Droop A., Hewinson J., Quail M.A., Iyer V., Shepherd R., Herbert R.A., Campbell P.J. (2020). The mutational signature profile of known and suspected human carcinogens in mice. Nat. Genet..

[35] Gillis S., Roth A. (2020). PyClone-VI: scalable inference of clonal population structures using whole genome data. BMC Bioinf..

[36] Shi K., Carpenter M.A., Banerjee S., Shaban N.M., Kurahashi K., Salamango D.J., McCann J.L., Starrett G.J., Duffy J.V., Demir Ö. (2017). Structural basis for targeted DNA cytosine deamination and mutagenesis by APOBEC3A and APOBEC3B. Nat. Struct. Mol. Biol..

[37] McCann J.L., Cristini A., Law E.K., Lee S.Y., Tellier M., Carpenter M.A., Beghè C., Kim J.J., Jarvis M.C., Stefanovska B. (2023). APOBEC3B regulates R-loops and promotes transcription-associated mutagenesis in cancer. bioRxiv.

[38] Xiao X., Yang H., Arutiunian V., Fang Y., Besse G., Morimoto C., Zirkle B., Chen X.S. (2017). Structural determinants of APOBEC3B non-catalytic domain for molecular assembly and catalytic regulation. Nucleic Acids Res..

[39] de Bruin E.C., McGranahan N., Mitter R., Salm M., Wedge D.C., Yates L., Jamal-Hanjani M., Shafi S., Murugaesu N., Rowan A.J. (2014). Spatial and temporal diversity in genomic instability processes defines lung cancer evolution. Science.

[40] Venkatesan S., Angelova M., Puttick C., Zhai H., Caswell D.R., Lu W.-T., Dietzen M., Galanos P., Evangelou K., Bellelli R. (2021). Induction of APOBEC3 exacerbates DNA replication stress and chromosomal instability in early breast and lung cancer evolution. Cancer Discov..

[41] DeWeerd R.A., Németh E., Póti Á., Petryk N., Chen C.-L., Hyrien O., Szüts D., Green A.M. (2022). Prospectively defined patterns of APOBEC3A mutagenesis are prevalent in human cancers. Cell Rep..

[42] Burns M.B., Lackey L., Carpenter M.A., Rathore A., Land A.M., Leonard B., Refsland E.W., Kotandeniya D., Tretyakova N., Nikas J.B. (2013). APOBEC3B is an enzymatic source of mutation in breast cancer. Nature.

[43] Harris R.S. (2015). Molecular mechanism and clinical impact of APOBEC3B-catalyzed mutagenesis in breast cancer. Breast Cancer Res..

[44] Damrauer J.S., Beckabir W., Klomp J., Zhou M., Plimack E.R., Galsky M.D., Grivas P., Hahn N.M., O’Donnell P.H., Iyer G. (2022). Collaborative study from the Bladder Cancer Advocacy Network for the genomic analysis of metastatic urothelial cancer. Nat. Commun..

[45] Helleday T., Eshtad S., Nik-Zainal S. (2014). Mechanisms underlying mutational signatures in human cancers. Nat. Rev. Genet..

[46] Wagener R., Alexandrov L.B., Montesinos-Rongen M., Schlesner M., Haake A., Drexler H.G., Richter J., Bignell G.R., McDermott U., Siebert R. (2015). Analysis of mutational signatures in exomes from B-cell lymphoma cell lines suggest APOBEC3 family members to be involved in the pathogenesis of primary effusion lymphoma. Leukemia.

[47] Chan K., Resnick M.A., Gordenin D.A. (2013). The choice of nucleotide inserted opposite abasic sites formed within chromosomal DNA reveals the polymerase activities participating in translesion DNA synthesis. DNA Repair.

[48] Doseth B., Visnes T., Wallenius A., Ericsson I., Sarno A., Pettersen H.S., Flatberg A., Catterall T., Slupphaug G., Krokan H.E., Kavli B. (2011). Uracil-DNA glycosylase in base excision repair and adaptive immunity. J. Biol. Chem..

[49] Krishnamurthy H., Danilovich N., Morales C.R., Sairam M.R. (2000). Qualitative and quantitative decline in spermatogenesis of the follicle-stimulating hormone receptor knockout (FORKO) mouse. Biol. Reprod..

[50] Simhadri S., Peterson S., Patel D.S., Huo Y., Cai H., Bowman-Colin C., Miller S., Ludwig T., Ganesan S., Bhaumik M. (2014). Male fertility defect associated with disrupted BRCA1-PALB2 interaction in mice. J. Biol. Chem..

[51] Wang H., Zhao R., Guo C., Jiang S., Yang J., Xu Y., Liu Y., Fan L., Xiong W., Ma J. (2016). Knockout of BRD7 results in impaired spermatogenesis and male infertility. Sci. Rep..

[52] Bailey M.H., Tokheim C., Porta-Pardo E., Sengupta S., Bertrand D., Weerasinghe A., Colaprico A., Wendl M.C., Kim J., Reardon B. (2018). Comprehensive characterization of cancer driver genes and mutations. Cell.

[53] Periyasamy M., Singh A.K., Gemma C., Kranjec C., Farzan R., Leach D.A., Navaratnam N., Pálinkás H.L., Vértessy B.G., Fenton T.R. (2017). p53 controls expression of the DNA deaminase APOBEC3B to limit its potential mutagenic activity in cancer cells. Nucleic Acids Res..

[54] Nikkilä J., Kumar R., Campbell J., Brandsma I., Pemberton H.N., Wallberg F., Nagy K., Scheer I., Vertessy B.G., Serebrenik A.A. (2017). Elevated APOBEC3B expression drives a kataegic-like mutation signature and replication stress-related therapeutic vulnerabilities in p53-defective cells. Br. J. Cancer.

[55] Zhang X., Wu Z., Hao Y., Yu T., Li X., Liang Y., Li J., Huang L., Xu Y., Li X. (2022). Aberrantly activated APOBEC3B is associated with mutant p53-driven refractory/relapsed diffuse large B-cell lymphoma. Front. Immunol..

[56] Roelofs P.A., Goh C.Y., Chua B.H., Jarvis M.C., Stewart T.A., McCann J.L., McDougle R.M., Carpenter M.A., Martens J.W., Span P.N. (2020). Characterization of the mechanism by which the RB/E2F pathway controls expression of the cancer genomic DNA deaminase APOBEC3B. Elife.

[57] Roelofs P.A., Timmermans M.A.M., Stefanovska B., Den Boestert M.A., Van Den Borne A.W.M., Balcioglu H.E., Trapman A.M., Harris R.S., Martens J.W.M., Span P.N. (2023). Aberrant APOBEC3B expression in breast cancer is linked to proliferation and cell cycle phase. Cells.

[58] Mayekar M.K., Caswell D.R., Vokes N.I., Law E.K., Wu W., Hill W., Gronroos E., Rowan A., Al Bakir M., McCoach C.E. (2023). The role of APOBEC3B in lung tumour evolution and targeted therapy resistance. bioRxiv.

[59] Roberts S.A., Lawrence M.S., Klimczak L.J., Grimm S.A., Fargo D., Stojanov P., Kiezun A., Kryukov G.V., Carter S.L., Saksena G. (2013). An APOBEC cytidine deaminase mutagenesis pattern is widespread in human cancers. Nat. Genet..

[60] Carpenter M.A., Li M., Rathore A., Lackey L., Law E.K., Land A.M., Leonard B., Shandilya S.M.D., Bohn M.-F., Schiffer C.A. (2012). Methylcytosine and normal cytosine deamination by the foreign DNA restriction enzyme APOBEC3A. J. Biol. Chem..

[61] Argyris P.P., Wilkinson P.E., Jarvis M.C., Magliocca K.R., Patel M.R., Vogel R.I., Gopalakrishnan R., Koutlas I.G., Harris R.S. (2021). Endogenous APOBEC3B overexpression characterizes HPV-positive and HPV-negative oral epithelial dysplasias and head and neck cancers. Mod. Pathol..

[62] Stenglein M.D., Burns M.B., Li M., Lengyel J., Harris R.S. (2010). APOBEC3 proteins mediate the clearance of foreign DNA from human cells. Nat. Struct. Mol. Biol..

[63] Beard C., Hochedlinger K., Plath K., Wutz A., Jaenisch R. (2006). Efficient method to generate single-copy transgenic mice by site-specific integration in embryonic stem cells. genesis.

[64] Bankhead P., Loughrey M.B., Fernández J.A., Dombrowski Y., McArt D.G., Dunne P.D., McQuaid S., Gray R.T., Murray L.J., Coleman H.G. (2017). QuPath: Open source software for digital pathology image analysis. Sci. Rep..

[65] Dobin A., Davis C.A., Schlesinger F., Drenkow J., Zaleski C., Jha S., Batut P., Chaisson M., Gingeras T.R. (2013). STAR: ultrafast universal RNA-seq aligner. Bioinformatics.

[66] Song L., Cohen D., Ouyang Z., Cao Y., Hu X., Liu X.S. (2021). TRUST4: immune repertoire reconstruction from bulk and single-cell RNA-seq data. Nat. Methods.

[67] Kim D., Paggi J.M., Park C., Bennett C., Salzberg S.L. (2019). Graph-based genome alignment and genotyping with HISAT2 and HISAT-genotype. Nat. Biotechnol..

[68] Trapnell C., Williams B.A., Pertea G., Mortazavi A., Kwan G., van Baren M.J., Salzberg S.L., Wold B.J., Pachter L. (2010). Transcript assembly and quantification by RNA-seq reveals unannotated transcripts and isoform switching during cell differentiation. Nat. Biotechnol..

[69] Rosenthal R., McGranahan N., Herrero J., Taylor B.S., Swanton C. (2016). deconstructSigs: delineating mutational processes in single tumors distinguishes DNA repair deficiencies and patterns of carcinoma evolution. Genome Biol..

[70] Bolger A.M., Lohse M., Usadel B. (2014). Trimmomatic: a flexible trimmer for Illumina sequence data. Bioinformatics.

[71] Chiang C., Layer R.M., Faust G.G., Lindberg M.R., Rose D.B., Garrison E.P., Marth G.T., Quinlan A.R., Hall I.M. (2015). SpeedSeq: ultra-fast personal genome analysis and interpretation. Nat. Methods.

[72] Blokzijl F., Janssen R., Van Boxtel R., Cuppen E. (2018). MutationalPatterns: comprehensive genome-wide analysis of mutational processes. Genome Med..

[73] Talevich E., Shain A.H., Botton T., Bastian B.C. (2016). CNVkit: genome-wide copy number detection and visualization from targeted DNA sequencing. PLoS Comput. Biol..

[74] Chen X., Schulz-Trieglaff O., Shaw R., Barnes B., Schlesinger F., Källberg M., Cox A.J., Kruglyak S., Saunders C.T. (2016). Manta: rapid detection of structural variants and indels for germline and cancer sequencing applications. Bioinformatics.

[75] Schwenk F., Baron U., Rajewsky K. (1995). A *cre* -transgenic mouse strain for the ubiquitous deletion of *loxP* -flanked gene segments including deletion in germ cells. Nucleic Acids Res..

[76] Serebrenik A.A., Argyris P.P., Jarvis M.C., Brown W.L., Bazzaro M., Vogel R.I., Erickson B.K., Lee S.-H., Goergen K.M., Maurer M.J. (2020). The DNA cytosine deaminase APOBEC3B is a molecular determinant of platinum responsiveness in clear cell ovarian cancer. Clin. Cancer Res..

[77] Takeo T., Nakagata N. (2011). Reduced glutathione enhances fertility of frozen/thawed C57BL/6 mouse sperm after exposure to methyl-beta-cyclodextrin. Biol. Reprod..

