# Supplementary material for: Human APOBEC3B promotes tumor development in vivo including signature mutations and metastases
Source: Cell Rep Med. 2023 Oct 4;4(10):101211. doi: 10.1016/j.xcrm.2023.101211 (PMC10591044; doi:10.1016/j.xcrm.2023.101211)
Supplement: Document S1. Figures S1–S7 [file mmc1.pdf]

Cell Reports Medicine, Volume *4*

Supplemental information

Human APOBEC3B promotes tumor development *in vivo* including signature mutations and metastases

Cameron Durfee, Nuri Alpay Temiz, Rena Levin-Klein, Prokopios P. Argyris, Lene Alsøe, Sergio Carracedo, Alicia Alonso de la Vega, Joshua Proehl, Anna M. Holzhauer, Zachary J. Seeman, Xingyu Liu, Yu-Hsiu T. Lin, Rachel I. Vogel, Rocio Sotillo, Hilde Nilsen, and Reuben S. Harris

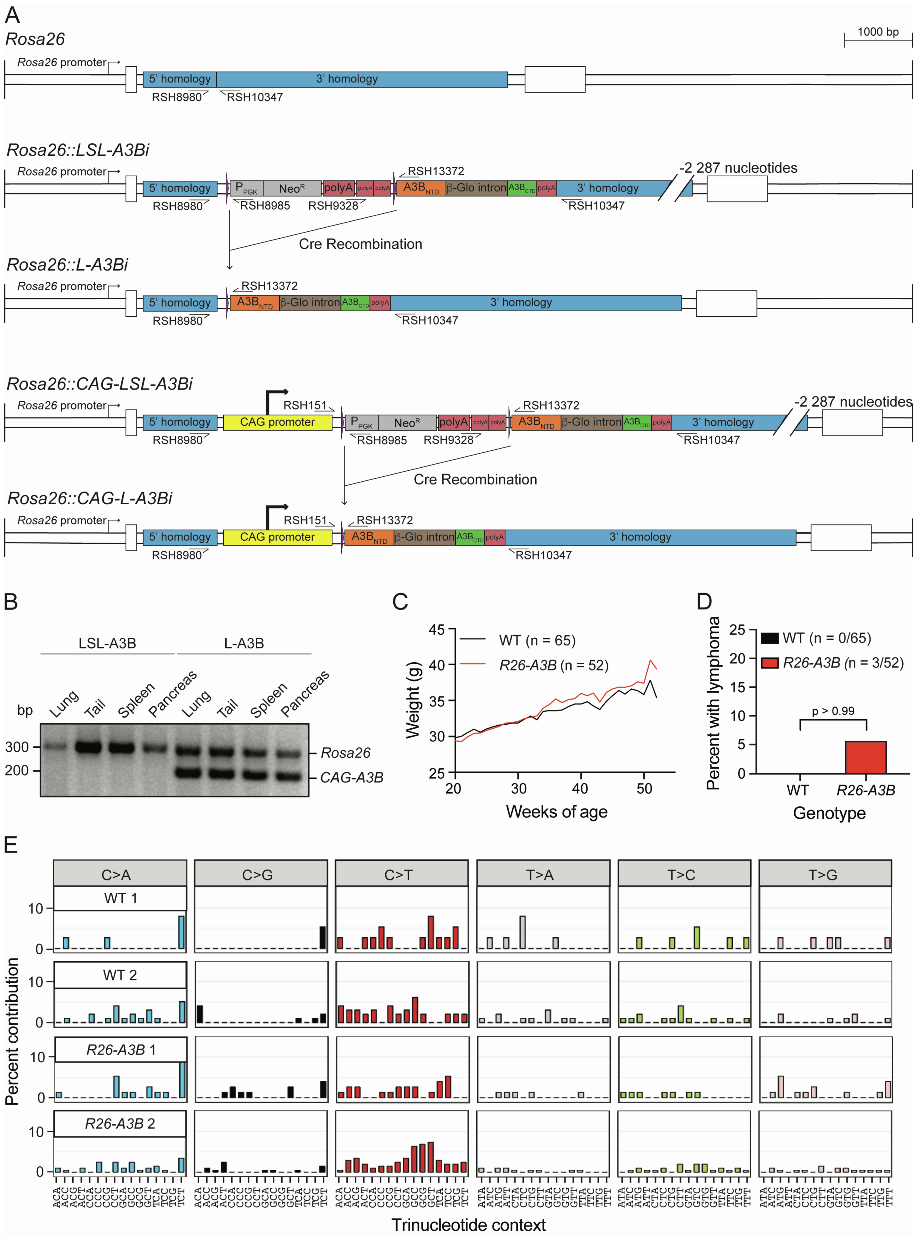

Figure S1. Construction and validation of mice expressing APOBEC3B, related to Figures 1 and 3

(**A**) To-scale schematics of the WT *Rosa26* locus and, following gene targeting, the fully intact *Rosa26::LSL-A3Bi* and *Rosa26::CAG-LSL-A3Bi* minigene alleles (i, intron). Cre-mediated recombination excises the *loxP*-STOP-*loxP* (LSL) transcriptional stop cassette and allows the *Rosa26* promoter or the *CAG* promoter to drive human *A3B* expression (abbreviated *R26-A3B* and *CAG-A3B*, respectively, here in this manuscript).

(**B**) PCR genotyping distinguishes the wildtype *Rosa26* locus from each knock-in allele using the indicated primer sets.

(**C**) Average of weights in grams taken weekly from 20-52 weeks of age for each indicated genotype. 65 WT mice and 52 *R26-A3B* mice are represented in this group.

(**D**) Percent of mice between 11 and 23 months that had a lymphoma upon euthanasia and necropsy (Fisher's exact test p-value indicated).

(**E**) Trinucleotide mutation profiles of the total SBS mutations in representative whole exome sequences from normal spleens of WT and *R26-A3B* mice, respectively.

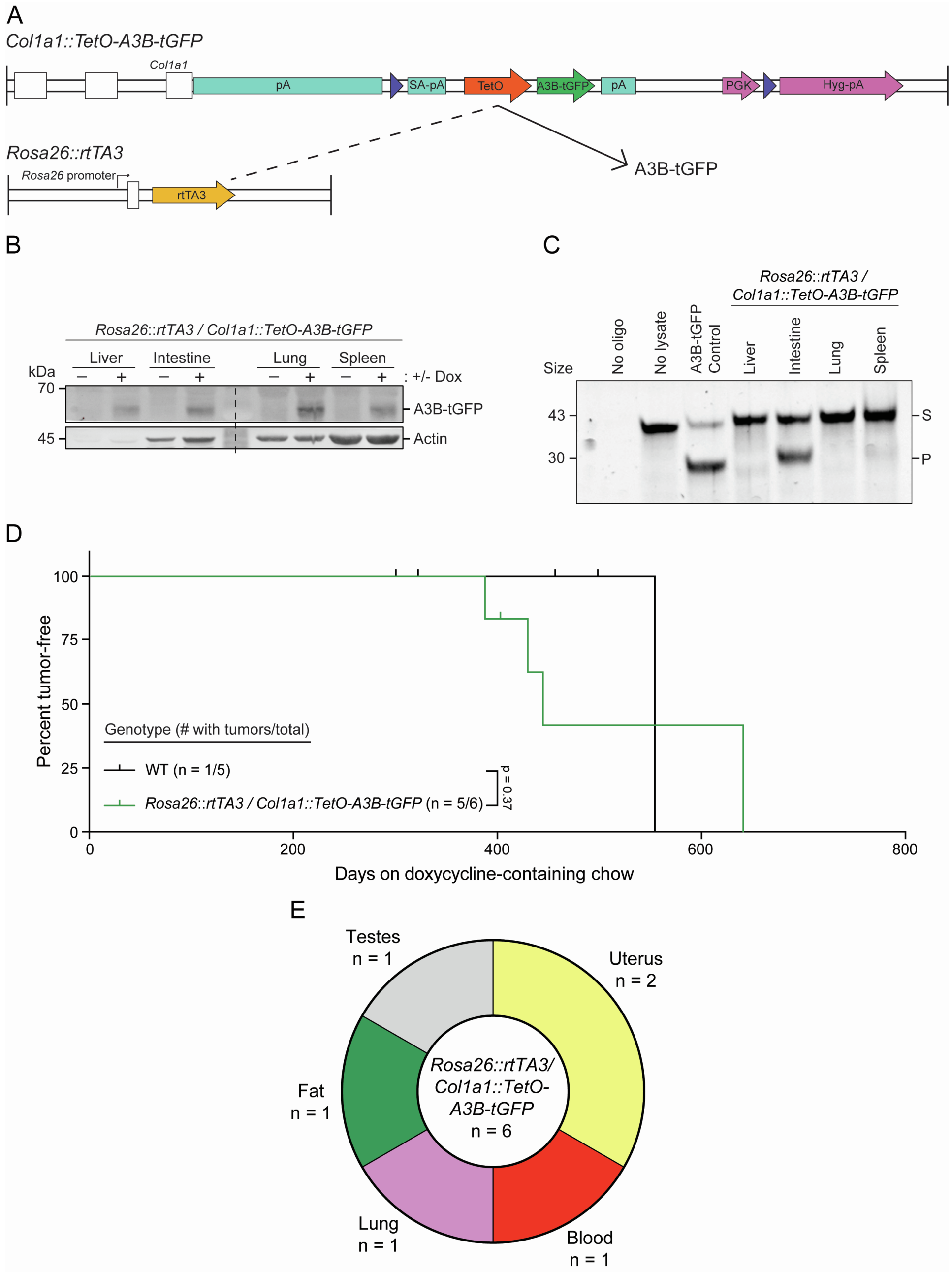

Figure S2. A3B expressed from the *Col1a1* locus is modestly tumorigenic, related to Figure 3

(**A**) Schematic of the engineered *Col1a1::TetO-A3B-tGFP* locus and *Rosa26::rtTA3* locus, which enables expression of A3B-tGFP following doxycycline (Dox) administration.

(**B-C**) Immunoblot and ssDNA deaminase activity of A3B-tGFP expressed in the indicated tissues from *Rosa26::rtTA3/Col1a1::TetO-A3B-tGFP* mice fed with (+) or without (-) Dox-containing chow for 15 days. 293T cells expressing A3B-tGFP were used as a positive control for activity (S, substrate; P, product), and actin provides a loading control.

(**D**) Kaplan-Meier curves comparing tumor-free survival of WT (n=5) and *Rosa26::rtTA3/Col1a1::TetO-A3B-tGFP* (n=6) animals fed with Dox-containing chow for 1-2 years from birth (log-rank Mantel-Cox test p-value indicated).

(**E**) Pie chart showing the total number of each tumor type found in Dox-treated *Rosa26::rtTA3/Col1a1::TetO-A3B-tGFP* mice.

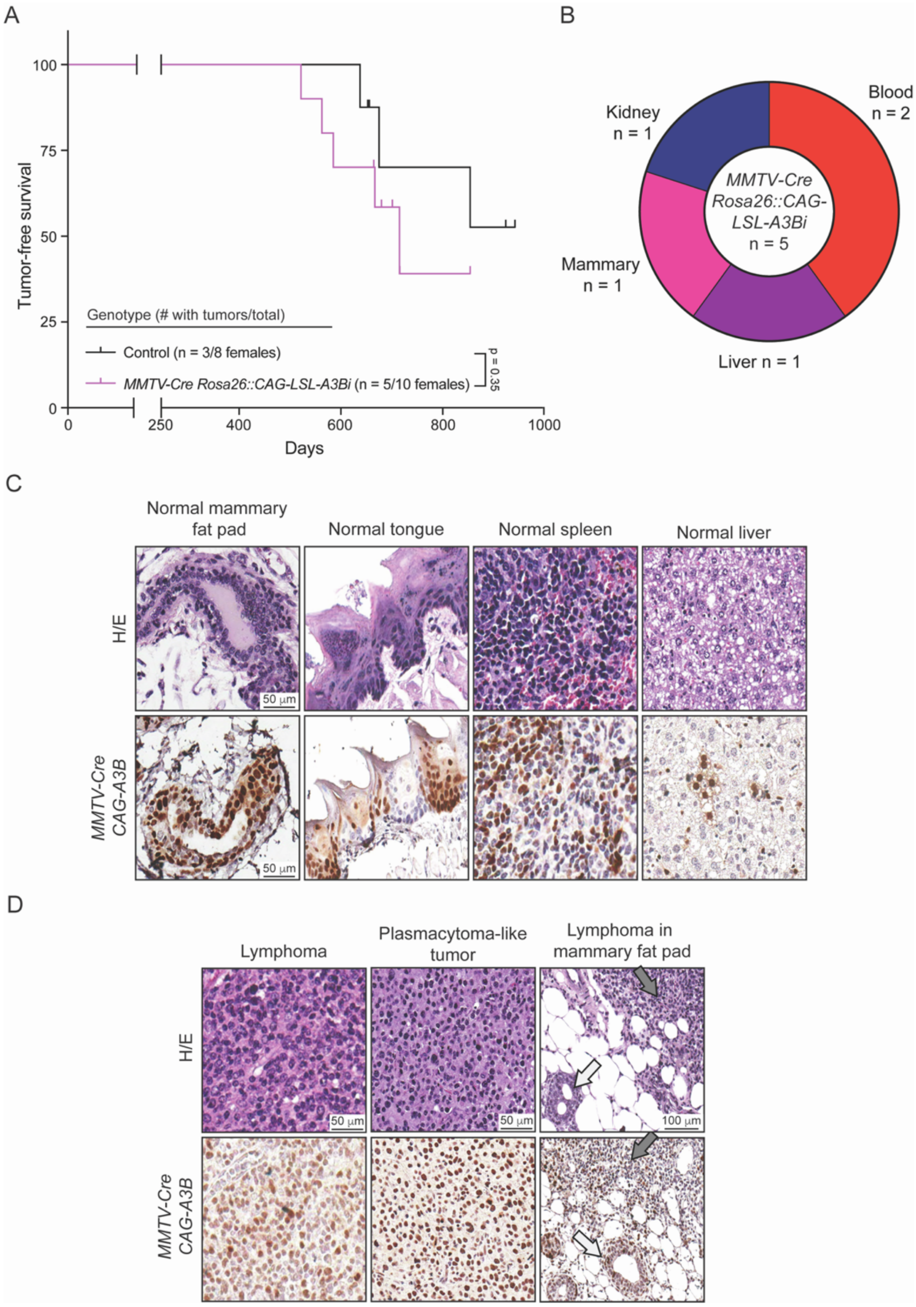

Figure S3. ***MMTV-Cre*** **mediated expression of** ***CAG-A3B*** **in select cell types has limited tumor phenotypes, related to Figure 3**

(**A**) Kaplan-Meier curve showing tumor-free survival of *MMTV-Cre* control (n=8 females) and *MMTV-Cre Rosa26::CAG-LSL-A3Bi* (n=10 females) mice (log-rank Mantel-Cox test p-value indicated).

(**B**) Pie chart showing the total number of each tumor type that developed in *MMTV-Cre Rosa26::CAG-LSL-A3Bi* females.

(**C**) H&E and anti-A3B IHC of indicated normal tissues in *MMTV-Cre Rosa26::CAG-LSL-A3Bi* mice. These immunohistological analyses indicate partial *MMTV-Cre* penetrance in multiple tissues beyond the mammary glands.

(**D**) H&E and anti-A3B IHC of representative lymphoproliferative tumors in *MMTV-Cre Rosa26::CAG-LSL-A3Bi* mice. Gray arrows point toward lymphomas, and white arrows to mammary glands.

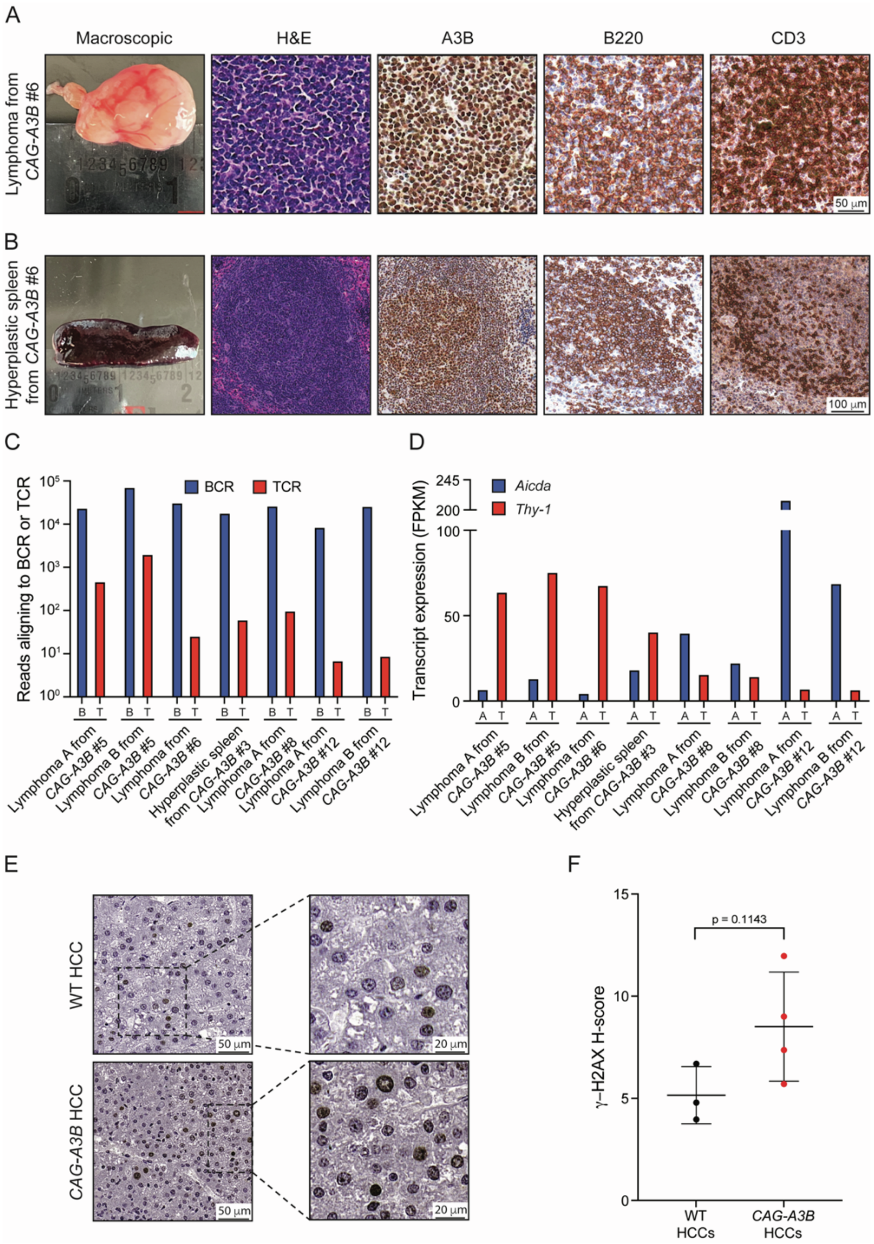

Figure S4. Phenotypic diversity of *CAG-A3B* tumors, related to Figure 4

(**A-B**) Macroscopic photograph and photomicrographs of H&E, anti-A3B, anti-B220, and anti-CD3 IHC of the lymphoma from *CAG-A3B* #6 and hyperplastic spleen from *CAG-A3B* #6, respectively. These lesions consist of a mixed B-cell and T-cell population, as shown by high levels of both B220 and CD3 immunostaining. High A3B levels are expressed within the nuclei of most cells. The macroscopic view of the lymphoma is also presented in Figure 4C.

(**C**) A histogram showing the number of *CAG-A3B* lymphoma RNAseq reads that align to recombined B-cell receptor (BCR; B) or T-cell receptor (TCR; T) genes using the TRUST4 algorithm. Each histogram bar represents the largest single contig for each recombined gene. The fact that the BCR rearrangement is clonal and represented by >100-fold more reads demonstrates that most lymphomas have a B cell origin.

(**D**) Transcript expression levels of B-cell specific transcript *Aicda* mRNA and pan T-cell marker *Thy-1* in lymphomas from *CAG-A3B* animals (additional analyses of the same RNAseq data sets as in panel C).

(**E-F**) Representative images and quantification of anti-g-H2AX immunostaining of HCCs from WT and *CAG-A3B* HCCs, respectively (mean +/- SD; Mann-Whitney U test p-value indicated).

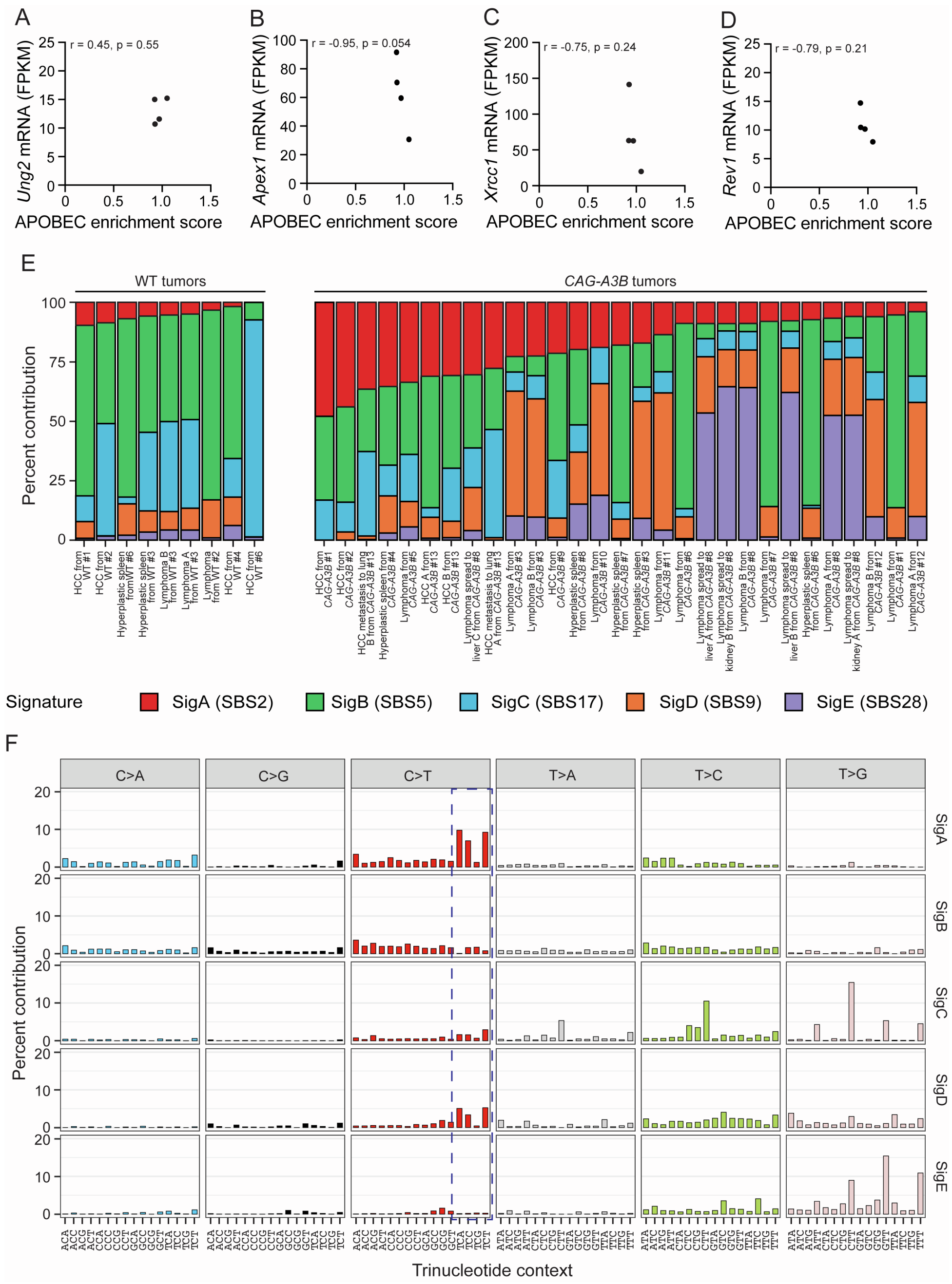

Figure S5. Additional mRNA expression and mutation signature analysis, related to Figure 5

(**A-D**) Scatterplots of APOBEC enrichment scores from WT lymphomas (n=4) compared to the mRNA levels of *Ung2*, *Apex1*, *Xrcc1*, and *Rev1*, respectively, from the same tumors (Pearson correlation coefficients and corresponding p-values indicated).

(**E**) Contribution of five *de novo* signatures to the overall tumor mutation landscape based on non-negative matrix factorization (n=29 *CAG-A3B*; n=9 WT). Upon comparison to COSMIC signatures, SigA is similar to SBS2 (cosine similarity, CS, = 0.79), SigB to SBS5 (CS = 0.93), SigC to SBS17 (CS = 0.94), SigD to SBS9 (CS = 0.74), and SigE to SBS28 (CS = 0.72).

(**F**) Trinucleotide mutation profiles of *de novo* signatures SigA to SigE extracted from tumors using non-negative matrix factorization.

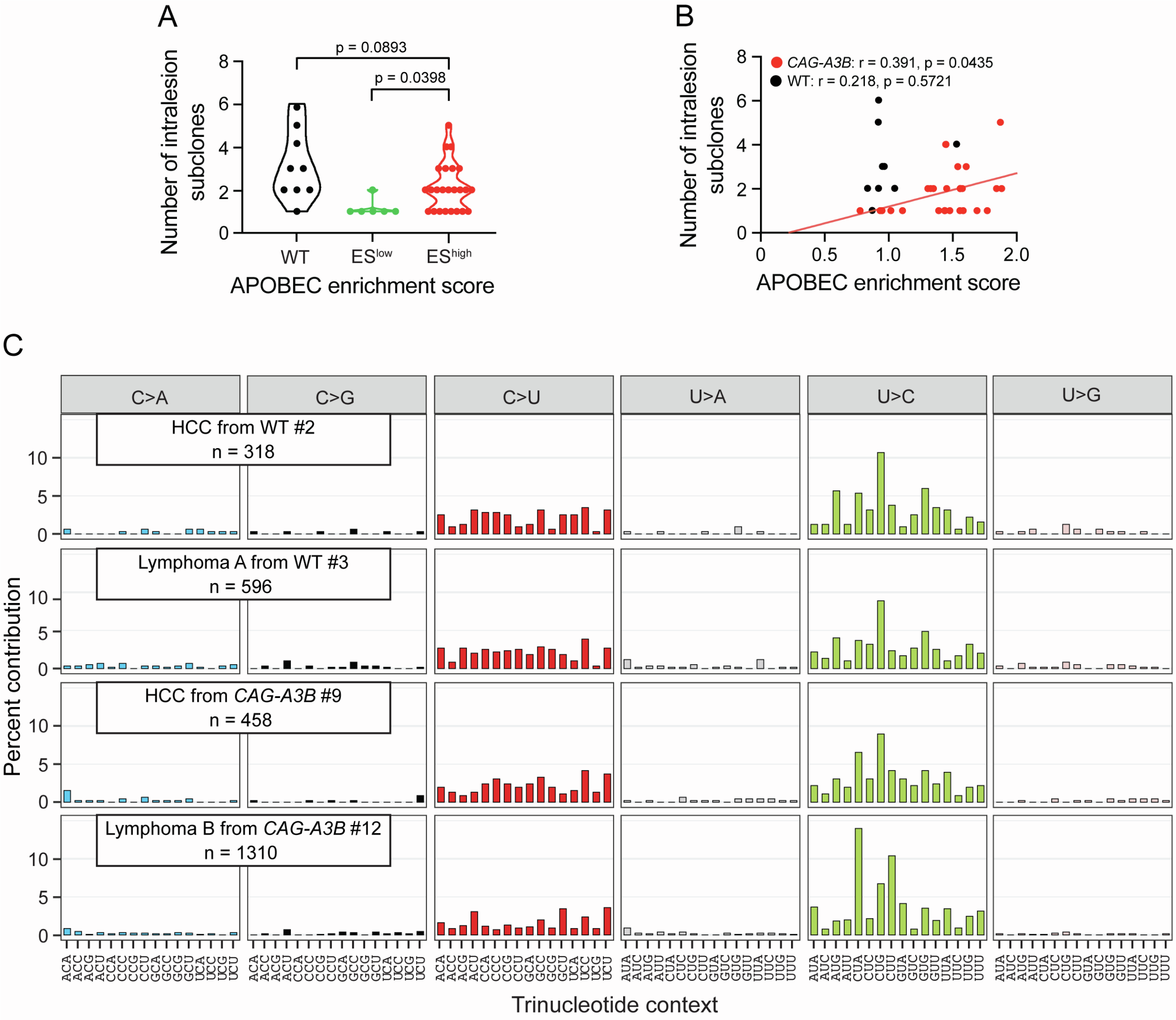

Figure S6. Analyses of intratumor heterogeneity and RNA editing, related to Figures 5 and 6
(**A**) Violin plots of the total number of intralesion subclones tumors as determined by PyClone-VI from WT mice in comparison to tumors from *CAG-A3B* animals with low or high APOBEC enrichment scores (ES; Mann-Whitney U test p-value indicated).
(**B**) Scatterplots comparing APOBEC mutation signature enrichment scores to the number of intralesion subclones in tumors from *CAG-A3B* and WT animals, respectively (linear regression line and Pearson correlation coefficients and p-values indicated).
(**C**) RNA editing profiles of representative tumors from WT or *CAG-A3B* animals (number of edits shown).

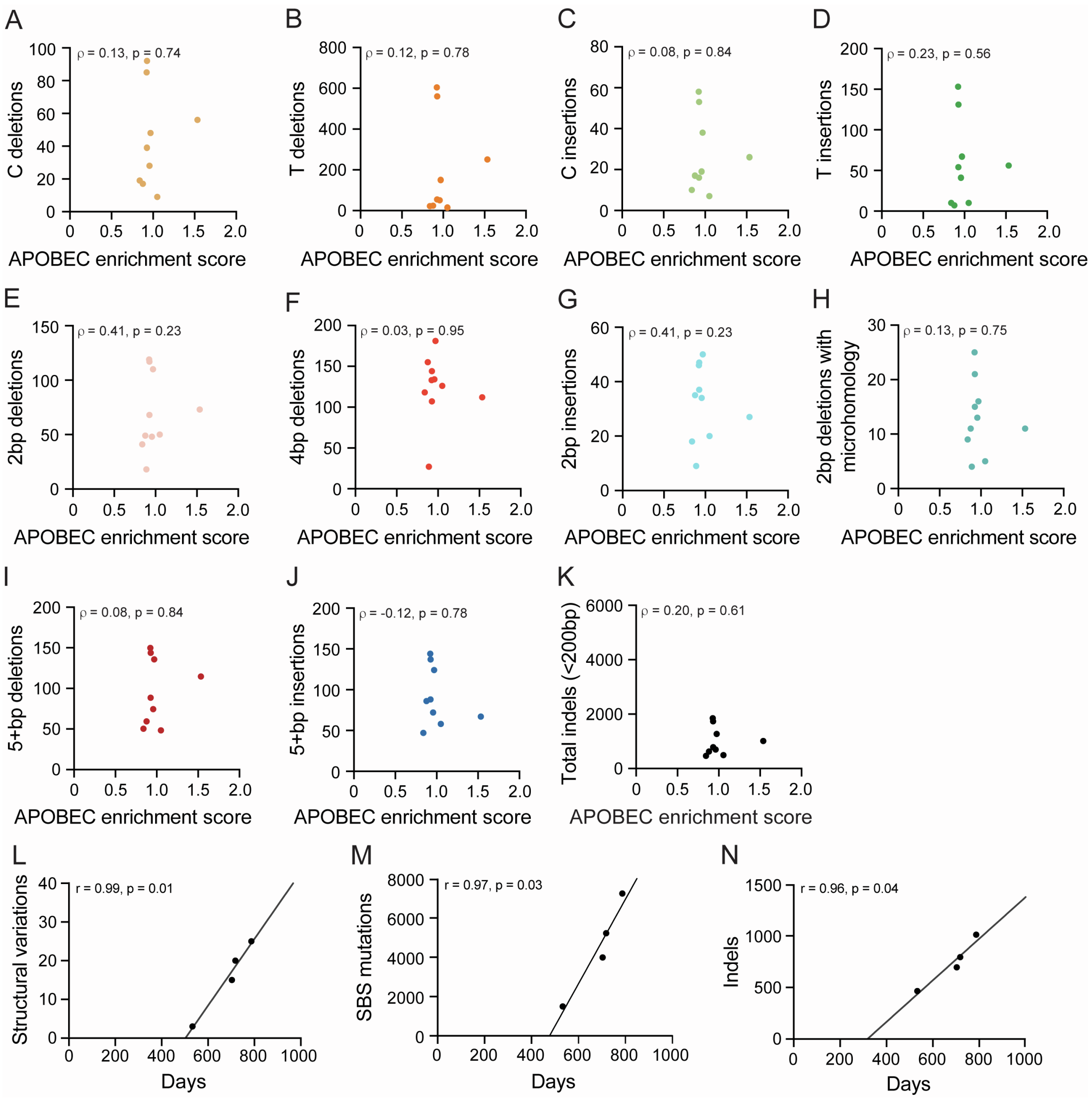

Figure S7. Genomic correlations in WT mice, related to Figures 5 and 7

(**A-J**) Scatterplots showing relationships between APOBEC mutation signature enrichment scores from WT tumors and the indicated indel types (Spearman's rank correlation coefficients and corresponding p-values indicated).

(**K**) Scatterplot showing the relationship between APOBEC mutation signature enrichment scores from WT tumors and the total number of indels <200bp in each tumor (Spearman's rank correlation coefficient and corresponding p-value indicated).

(**L-N**) Scatterplot of the number of structural variations, SBS mutations, and indels in HCCs (n=4) from WT animals in comparison to age of the animal at the time of sacrifice (linear regression lines and Pearson correlation coefficients and corresponding p-values are shown).
